# Stratification of malaria incidence in Papua New Guinea (2011–2019): Contribution towards a sub-national control policy

**DOI:** 10.1371/journal.pgph.0000747

**Published:** 2022-11-21

**Authors:** Osama Seidahmed, Sharon Jamea, Serah Kurumop, Diana Timbi, Leo Makita, Munir Ahmed, Tim Freeman, William Pomat, Manuel W. Hetzel

**Affiliations:** 1 Swiss Tropical and Public Health Institute, Allschwil, Switzerland; 2 Papua New Guinea Institute of Medical Research, Goroka, Papua New Guinea; 3 University of Basel, Basel, Switzerland; 4 National Department of Health, Port Moresby, Papua New Guinea; 5 Rotarians Against Malaria, Port Moresby, Papua New Guinea; Tulane University School of Public Health and Tropical Medicine, UNITED STATES

## Abstract

Malaria risk in Papua New Guinea (PNG) is highly heterogeneous, between and within geographical regions, which is operationally challenging for control. To enhance targeting of malaria interventions in PNG, we investigated risk factors and stratified malaria incidence at the level of health facility catchment areas. Catchment areas and populations of 808 health facilities were delineated using a travel-time accessibility approach and linked to reported malaria cases (2011–2019). Zonal statistics tools were used to calculate average altitude and air temperature in catchment areas before they were spatially joined with incidence rates. In addition, empirical Bayesian kriging (EBK) was employed to interpolate incidence risk strata across PNG. Malaria annual incidence rates are, on average, 186.3 per 1000 population in catchment areas up to 600 m, dropped to 98.8 at (800–1400) m, and to 24.1 cases above 1400 m altitude. In areas above the two altitudinal thresholds 600m and 1400m, the average annual temperature drops below 22°C and 17°C, respectively. EBK models show very low- to low-risk strata (<100 cases per 1000) in the Highlands, National Capital District and Bougainville. In contrast, patches of high-risk (>200 per 1000) strata are modelled mainly in Momase and Islands Regions. Besides, strata with moderate risk (100–200) predominate throughout the coastal areas. While 35.7% of the PNG population (estimated 3.33 million in 2019) lives in places at high or moderate risk of malaria, 52.2% (estimated 4.88 million) resides in very low-risk areas. In five provinces, relatively large proportions of populations (> 50%) inhabit high-risk areas: New Ireland, East and West New Britain, Sandaun and Milne Bay. Incidence maps show a contrast in malaria risk between coastal and inland areas influenced by altitude. However, the risk is highly variable in low-lying areas. Malaria interventions should be guided by sub-national risk levels in PNG.

## Introduction

Despite global efforts to eradicate malaria, the World Malaria Report 2021 reveals stalled (if not failed) progress in recent years [1, 2]. In Papua New Guinea (PNG), malaria remains a leading cause of morbidity and mortality with an estimate of 1.5 million cases in 2020 representing 86% of the disease burden in the Western Pacific Region [1]. A plethora of microcosms influenced by rugged terrain and limited transport infrastructure makes malaria a highly heterogeneous problem in PNG [3, 4]. The most common species, *Plasmodium falciparum* and *P*. *vivax*, rapidly adapt to changes in the environment that result in differential responses to control measures [5–8]. Furthermore, eleven mosquitoes belonging to *the Anopheles punctulatus* group complement one another’s niche in malaria transmission. The highly heterogeneous malaria transmission risk between and within the regions of PNG is a challenge for implementing effective control.

Since 2004, the Global Fund to Fight AIDS, Tuberculosis and Malaria (GFATM) has supported PNG to scale-up malaria control [9–12]. The national malaria control programme (NMCP) delivers key interventions both at village and health facility levels. These include long-lasting insecticidal mosquito nets (LLINs), malaria Rapid Diagnostic Tests (mRDTs), Artemisinin-based Combination Therapy (ACT) and campaigns of Behaviour Change Communication (BCC) [13]. Also, mobilisation of resources has involved capacity building and strengthening the management of the NMCP [14, 15].

Since 2006, LLINs have been distributed in PNG, mainly in areas below 2000m. Due to limitation of funding and perceived low risk of malaria at altitudes 1600-2000m, the NMCP have recently stopped distribution of nets in areas at these altitudes (Rotarians Against Malaria (RAM), personal communication, 2021). This policy shift has mainly concerned the population living in the Highlands Region. Although malaria is not a major public health problem in the Highlands, decision-making should consider local settings where the environment is receptive and seasonal or epidemic malaria transmission may occur [16]. Indeed, local outbreaks could be devastating for unprotected people who lack immunity to the disease. Therefore, stratification of the risk of malaria at a micro-spatial scale could help to allocate limited resources efficiently for maximum impact.

Previous epidemiological studies considered coastal lowlands holo-endemic for malaria, while highland areas contained epidemic-prone and non-malarious areas [11]. Early work in 1973 divided the country into five risk strata: hypo, hypo-to-meso, meso, hyper and holoendemic [17]. Broadly speaking, at that time, hyper-endemicity prevailed in coastal areas while mountainous areas were mainly hypo-endemic. Later, a micro-stratification exercise in the Highlands and Momase Regions (2001–2005) reported differences in the composition of strata between the two regions and within their provinces [3, 18–22]. However, it is unclear how the scale-up of malaria control interventions has affected these strata.

Over the last two decades, advances in geospatial technology have allowed mapping of malaria risk at sub-national scale [23–27]. In particular, geostatistical techniques such as kriging are used to predict the disease risk at continuous surface based on autocorrelation of samples measured in nearby sites. Malaria cases reported by the routine health system were used in several studies to estimate malaria incidence or combined with prevalence to map the disease risk using geostatatical methods [28–31]. In a highly heterogeneous landscape, a Bayesian kriging method could optimize the interpolation result by dividing the extensive surface into small parts to model and calibrating localized models using simulations to fit their parameters [32–34]. However, mapping of malaria risk at sub-national level requires collection of more extensive data representative to local transmission settings.

Routine surveillance data collected by health facilities can be useful to stratify malaria risk in the facilities’ catchment areas. In PNG, healthcare is delivered via a decentralised system involving: a national referral hospital, provincial and district hospitals, health centres, sub-health centres, community health posts and aid posts. The primary delivery unit of public health services to local communities is the health centre and sub-health-centre. These health facilities constitute the base of the National Health Information System (NHIS). NHIS data is comprehensive, covering all registered health facilities in the whole country. There is ongoing transition of NHIS reporting at health facility level from paper-based to electronic format [35].

This study investigates malaria risk factors in PNG at the level of catchment areas of health facilities and stratifies interpolated incidence over the whole country to support the sub-national targeting of malaria control interventions.

## Methodology

### Study area

PNG is divided into four regions (Highlands, Islands, Momase and Southern), and administratively into 22 provinces including the Autonomous Region of Bougainville, and the National Capital District (NCD). A centrally ridge of mountains running from the northeast to southwest and intervening valleys characterise the mainland of PNG. The altitude in the Highlands Region exceeds 4,500 m above sea level, while there are also mountainous areas above 2000 m altitude in the other regions. Large plains can be found in Southern and Momase regions along the rivers Fly, Sepik, and Ramu, see S1 Fig.

### Datasets

#### Location of health facilities

An updated list of health facilities (HFs) was acquired from the National Department of Health (NDoH). The list contained 808 HFs (varied in their functioning status between the years) excluding aid posts, with their codes and type. Geographical locations of HFs were obtained from three sources: 1) a geocoded dataset from a health facility survey conducted by the Papua New Guinea Institute of Medical Research (PNGIMR) in 2014; 2) a United Nations Development Programme (UNDP) excel sheet with latitude and longitude of HFs available online on the website Humanitarian Data Exchange(HDX) for 2018 [36]; 3) Shapefiles extracted from maps generated by RAM for data collected during LLINs distribution campaigns. Hence, a master sheet was compiled, corrected for duplications and codes of NDoH. Google Earth was further used to check and confirm the locations of HFs.

#### Population distribution

Geocoded data of census units (wards/villages) were obtained for the 2011 National Population and Housing Census, published by the National Statistical Office (NSO) of PNG. This dataset contained 27,000 census units but with over 10% of duplicates in their geolocations. In addition, we obtained from HDX a high-resolution population density map developed by Facebook in partnership with Columbia University [37]. In the generation of this dataset, Convolutional Neural Networks were used to combine high-resolution satellite images with the best available census data (for further details on methodology, see https://dataforgood.fb.com). The Facebook population density map for PNG contains the geo-coordinates of 691,933 residential buildings/places and associated estimates of the overall population in 2015.

### Malaria cases reported by NHIS

Annual NHIS malaria reports for the years 2011 to 2019 were obtained from NDoH. The excel sheets contained aggregate annual numbers of confirmed malaria cases by health facility and according to the diagnostic method used (mRDT vs. microscopy), age groups (children under five, adolescents 5–14 years, and adults), sex (males and females), and *Plasmodium* species. Besides, numbers of reports received per year, numbers of presumptive cases defined as inpatients and outpatients prescribed ACT but not tested for infection are provided in these sheets. According to NHIS, HFs are responsible for aggregating the daily malaria register data reported at their premises and dependent aid posts within the catchment. Paper-based forms are submitted to the Provincial Health Office. There, the latter is responsible for entering the summarised reports in the NHIS electronic forms. Further, crosschecks, including cleaning and re-entry are done at the national level for quality control [35].

#### Elevation raster of PNG

Tiles of elevation raster for PNG were retrieved from the Global 3D Digital Elevation Model TanDEM-X (for further details, see https://www.dlr.de). The elevation dataset, developed by the German Aerospace Center (DLR), has a high resolution of 90x90 meters with a height accuracy of one meter. Previously, two radar satellites (TanDEM-X and TerraSAR-X) were used to capture images of the earth’s surface for four years (2011–2015) but from different angles. Further, DLR had processed and resampled the original TanDEM-X images (of 12m resolution) to create the current version of TanDEM-X. Raster tiles were collated and clipped for the geographical area of PNG before the merged raster was corrected for the elevation of water bodies and bordering lines using ArcGIS Desktop 10.5.

*The friction surface of PNG*. A raster of global friction surface was obtained from the Malaria Atlas Project (MAP) website to estimate the travel time between human settlements and health facilities [38]. Initially, MAP rasters with a resolution of 1x1 km were constructed by merging Open Street Map (OSM) with a distance-to-roads database obtained from Google in November 2016 and March 2016. A country-level raster was extracted for PNG using the clipping tool of ArcGIS. The pixel values represent speeds of movement, i.e., minutes required to travel one meter, for which the fastest mode (e.g., a motorized vehicle, foot or boat) between the two datasets was given precedence.

*Climatic data of PNG*. WorldClim is a set of global gridded data that contains layers of average monthly climatic variables for the period 1970–2000. Monthly averages of air temperature, vapour pressure and rainfall were retrieved from WorldClim Version 2.3 [39]. This source includes gridded raster maps with a spatial resolution of one km^2^. In ArcGIS, climatic maps were clipped to the country area and corrected for water bodies and physical boundaries in PNG.

### Analytical methods

#### Distribution of population by altitude and temperature suitability for malaria

For this task, we added an external tool: "Zonal Statistics as Table 2" to the Spatial Analysis toolset of ArcGIS to prevent overlapping polygons when calculating catchments’ areal altitude. Averages of the altitude of census units were calculated in buffers of a six km radius using the zonal statistics tool of ArcGIS. The cut-off of the buffer was based on the travel time chosen in the delineation of the catchment area (see “Delineation of catchment areas”). Increments of 200m were used to show percentages of people and households living at specific altitudes.

The elevation raster of PNG was resampled to a resolution of one km^2^ to ensure correspondence in the resolution of WorldClim temperature and TanDEM-X elevation. The zonal statistics tool was used to associate average altitude with grids of annual temperature (1 km^2^). The raster files of elevation and mean annual temperature were converted to points feature and grid centres.

The residential places belonging to each census ward were intersected with raster pixels of WorldClim (1970–2000) before the average air temperature (T) was calculated and used in extrinsic incubation period (EIP) models. We utilized two degree-days models described in [40–42] to calculate EIP for *P*. *falciparum* and *P*. *vivax*:

$${EIP}_{falciparum}(days)=\frac{105(degree.days)}{T-14.5}$$

$${EIP}_{vivax}(days)=\frac{111(degree.days)}{T-16}$$

*Accessibility map of health facilities*. The raster of friction surface of PNG was updated with Open Street Map (OSM) 2020 data to account for existing roads and extensions reported after the release of the MAP dataset 2015. OSM road vectors of PNG were downloaded, rasterised into 1 km^2^ pixels and merged with the MAP friction raster using ArcGIS. Averages of travel time to cross raster pixels of different road types were used to generate a lookup table and update pixel values corresponding to OSM roads in the friction map.

To generate an accessibility map of HFs, we used the Cost Distance tool of ArcGIS to identify the nearest health facilities to human settlements and calculate their travel time. The method uses a least-cost-path algorithm to cumulate the cost of crossing raster pixels (/minute) between the source (i.e., human settlements) and destination cells (i.e., health facilities), according to the following equation:

$$d_{i}({HF}_{j})=\underset{i}{\min}\sum_{i=1}^{n}c_{i-1}+\beta \frac{\alpha_{1}+\alpha_{2}}{2},\;\left\{ \begin{array}{l} \;\beta =1,\;∠=180{}^{\circ}\\ \beta =1.414214,\;∠=90{}^{\circ} \end{array}\right.$$

Where: *d*_*i*_(*j*) = least cost of travel from a human settlement i to HF j, c = minimum cost of travel taken over all neighbours of i, α = travel cost to cross between centres of cell 1 and cell neighbour 2, β = a constant dependent on the link angle between the two cells (perpendicular or diagonal).

Hence, different routes are tested before a path with the shortest travel time is determined. The ArcGIS tool produces two separate raster maps: 1) travel cost to nearest HF, and 2) allocation index of nearest health facility for each raster pixel. The pixel values corresponding to human settlements were extracted while a table containing travel time and index of nearest health facility was generated.

*Delineation of catchment areas*. The map of travel time to the nearest HF was used in the delineation of catchment areas. We assumed that treatment seekers would only visit the nearest HF or pool of HFs reachable within a specific threshold of travel time. Accessibility threshold of two hours was used to delineate the catchment areas of HFs. The selection of the two hours was based on previous health facility surveys in PNG, which show varied but high travel-time across the regions ranging between 43–88 minutes [43]. In addition, meetings with health providers at HFs during the Malaria Indicator Survey 2019/20 suggested that a person seeking care would travel a maximum of two hours to reach a nearby health facility. If there is no HF within two hours we assumed patients do not seek treatment at a health facility.

*Calculation of population size in catchment areas*. Instead of using the census 2011, we spatially joined census units with the Facebook raster map. Growth rates of NSO for each province were used to project the population for every residential place in the years 2012–2020. A bottom-up approach was used to calculate the total population living in the catchment areas, provinces, and country. The catchment population in the human settlements pixels identified in the delineation process—projected from census 2011 data—was summed up.

Further, we assumed that the same pool of treatment seekers shares HFs within a travel time of one hour. Hence, catchment populations were summed up before being evenly divided between the sharing HFs. In addition, we calculated the total numbers of people living in each stratum and province by spatially joining the human settlements with a vector feature converted from the raster map.

*Malaria incidence at catchment areas of HFs*. ***Adjustment of reported cases*.** Monthly malaria cases (both presumptively diagnosed but not tested cases and confirmed cases using 245 light microscopy or mRDT diagnosis) reported by HFs were used to calculate malaria incidence. Only HFs with at least 12 monthly reports during the nine years (2011–2019) were included in the analysis. In addition, presumptive cases were adjusted using positivity rates specific to province-year:

$$N_{xm}=K_{xm}*\varepsilon_{x}+C_{xm}$$

Where: N_xm_ = adjusted reported cases at HF x in month m, K_xm_ = monthly clinical cases reported at HF, *ε* = positivity% of tested cases calculated at province-year for the HF x, *C*_*xm*_ = monthly confirmed cases reported at the HF.

***Estimation of patients unseeking care at HFs*.** To account for under reporting at HFs considering spatial variation in treatment-seeking, we estimated patients unseeking care in catchment areas relative to reported ones at HFs. For this purpose, weighted mean was calculated using proportions of patients with fever that sought treatment at a HF reported in the previous three MIS 2013/14, 2016/17 and 2019/20 [12, 44, 45]. Due to small numbers of respondents at a province level, the proportion of treatment-seekers was calculated at a regional level (i.e. Islands, Highlands, Momase, and Southern). Next, estimations of patients unseeking care were adjusted using positivity rates as above:

$$U_{xm}=(1-\alpha_{x})*\varepsilon_{x}*N_{xm}$$

Where: U_xm_ = adjusted estimate of malaria cases unseeking care at HF x in month m, *α*_*x*_ = proportion of treatment-seeking proportion calculated at region-year for the HF x.

The adjusted reported cases and estimates of patients unseeking care of specific HF x, were summed:

*T*_*xm*_ = *N*_*xm*_+*U*_*xm*_ Where: T_xm_ = sum of reported cases and estimate of patients unseeking care at HF x in month m.

The average annual incidence per 1000 in a catchment of a health facility was calculated for the period 2011–2019 using the following equation:

$$I_{xy}=\frac{\sum_{m=1}^{q}T_{xm}}{P_{xy}\times q_{xy}}\times 12\times 1000$$

Where: I_xy_ = average annual incidence rate of HF "x" in year "y", P_xy_ = projected population in a catchment of HF x in the year y, q = total of reported months in year y and health facility x.

District-specific growth rates provided by the NSO were used to project the population of a catchment in a specific year using the following growth formula:

$$P_{xy}=P_{x0}e^{r_{n}t}$$

Where: P_xy_ = the projected population of catchment x at year y, P_x0_ = the population at catchment area x in the census year 2011, r_n_ = growth rate specific to n district where the catchment area x lie (/year), t = difference between the projected and census year (y-2011).

Further, populations of specific age groups and sex were estimated, assuming that their proportions in the census year 2011 remain constant. The spatial joining tool of ArcGIS was used to link the locations of HFs with the NHIS excel sheets of cases and with the projected population living in the catchments.

Boxplots on average incidence rates at HFs against altitude of catchment areas were generated using R. While solid boxes were used to span the interquartile range (i.e., Q1 to Q3), the segment line inside each boxplot indicated the median value over the nine years. The solid lines (whiskers) were extended to include roughly 99% of data points. The upper whisker is at the minimum of Error bars = +1.5* Interquartile range (IQR).

#### Catchment areas with few malaria cases among children under 15 years

The purpose of this analysis it to use malaria among children—who are expected to be less mobile—as a proxy to measure local transmission. To identify catchment areas with limited malaria cases among children and adolescents (2011–2019), we purposefully selected HFs with the following criteria: i) average monthly number of confirmed cases is less than one case per reported month; ii) more than 100 individuals were tested using microscopy or mRDTs; iii) proportion of positive cases below 15 years among all positive cases is less than 30%.

Population size in these catchment areas and average annual incidence rates among the general population were calculated and grouped by altitude. The total number of LLINs issued in these catchment areas by distribution campaigns (2011–2019) was summed up.

#### Modelling of malaria risk strata

We employed empirical Bayesian kriging (EBK) in ArcGIS to interpolate malaria risk between catchment areas across PNG. A general assumption in kriging is spatial dependence between proximal features more than distal ones. In kriging methods, a semivariogram—which is a function describing the relationship between the distance and half the average squared difference of the values for pairs of locations—is used. To estimate the values in unsampled locations, weights based on the semivariogram are used in parameterization of a prediction model [33].

EBK is a geostatistical technique that uses an iterative process of subsetting and simulations to model best estimates in non-sampled locations. The process starts by estimating a semivariogram for each subset of observations. Then, the semivariogram is used in unconditional simulation of new (artificial) data. This is a loop step repeated for defined number of times (i.e., previous dataset used to generate a new semivariogram used to estimate new dataset, over and over). The output of this iterative process is a large set of semivariograms plotted together to estimate a final empirical semivariogram fitting the distribution [32, 33, 46, 47].

EBK employs a restricted maximum likelihood (RML) methodology to optimise the model parameter [32, 33]. Spectrums of semivariograms in the neighbourhood of a prediction location are sampled probabilistically to get a likelihood value. Hence, unmeasured locations can be interpolated using weighted, measured values, according to the following equations:

$${\hat{Z}}_{\left(s_{0}\right)}=\sum_{i=1}^{N}\lambda_{i}{\overset{´}{Z}}_{\left(s_{i}\right)}|Z,\theta_{i}$$

$$\lambda_{i}=\frac{f(Z|\theta_{i})}{\sum_{i=1}^{N}f(Z|\theta_{i})}$$

Where: $\overset{´}{Z}(s_{i})$ = simulated value at location i, Z = measured values, λ_i_ = weight for measured value at location i, *θ*_*i*_ = the model parameters, s_0_ = the prediction location, N = number of observed sites.

Unlike other kriging methods, EBK allows a more accurate estimation of standard error based on a set of semivariograms instead of a single one [32]:

$$\sigma_{z(s_{0})}^{2}|Z=\sum_{i=1}^{N}\lambda_{i}\left(\sigma_{z(s_{0})}^{2}|Z,\theta_{i}+{\left(Z_{s_{0}}|Z,\theta_{i}-{\hat{Z}}_{\left(s_{0}\right)}\right)}^{2}\right)$$

Where: $\sigma_{z(s_{0})}^{2}$ = variance of prediction Z at location s_0_. For more details on algorithms of EBK, see [32, 47].

Two risk maps were generated using the EBK tool in the Geostatistical Analyst of ArcMap. The input point features were the average annual incidence of malaria (2011–2019) using two data sets of malaria cases (i.e., adjusted presumptive cases + confirmed cases + estimates of patients unseeking care) among: (1) the general population, and (2) children and adolescents below 15 years. With an overlap factor of 1.4, semivariograms for subsets of 30 catchment areas were simulated and automatically adjusted parameters. Cell size in raster maps was set to one km^2^. A mask of PNG country boundaries was used as a geographical extent to clip the risk maps.

Four strata of average annual incidence per 1000 population, adjusted to the interpolated surface, were depicted in the risk maps: very low, low, moderate, and high. We applied PNG-specific cut-offs based on the frequency distribution of incidence values. Hence, the cut-offs of cases per 1000 chosen for the strata are: <30 as very low, 30–100 as low, 100–200 as moderate, and >200 as high. The cut-offs proposed in the World Health Organization’s Framework for malaria elimination were not sufficiently discriminative in PNG [48].

*Cross-validation of the models*. A Leave-One-Out-Cross-Validation approach (LOOCV) was used. Hence, the entire dataset was employed to verify the model performance by successively removing data points, one at a time, and evaluate the predicted value based on other neighbouring data points in the searching window. In ArcGIS, The Geostatistical Analyst tool automatically generated five statistics of cross-validation for EBK models: mean error (ME), root-mean-square error (RMSE), average standard error (ASE), mean standardised error (MSE), and root-mean-square standardised error (RMSSE) [49] For more details on equations of these statistics, see S1 Text.

A good fit EBK model will have the following criteria: 1) an ME nearly zero; 2) small and similar values of RMSE and ASE; and 3) an RMSSE error close to one. Hence, we experimented for optimal models by tuning up EBK parameters to obtain useful cross-validation statistics independently for incidence rates among the general population and children below 15 years. Further, predicted values were extracted at observations locations and a correlation coefficient (r) was calculated.

## Results

### Climatic suitability for malaria

Across PNG, mean annual temperature (for the period 1970–2000) in residential places ranged from 6.4 to 27.8°C, while mean annual precipitation varied from 1,202 mm to 6,619 mm. The large difference in temperature between coastal and inland areas on the mainland is primarily attributable to altitude, above all in the central mountain range that reaches over 4500 meters.

In general, the relationship between altitude and temperature in PNG (R^2^ = 0.985, RMSE = 5.09) demonstrates that for every 200 m rise in altitude, air temperature drops roughly one degree Celsius. Annual averages and seasonal changes in temperature and precipitation are shown in S3 Fig.

**Fig 1:**
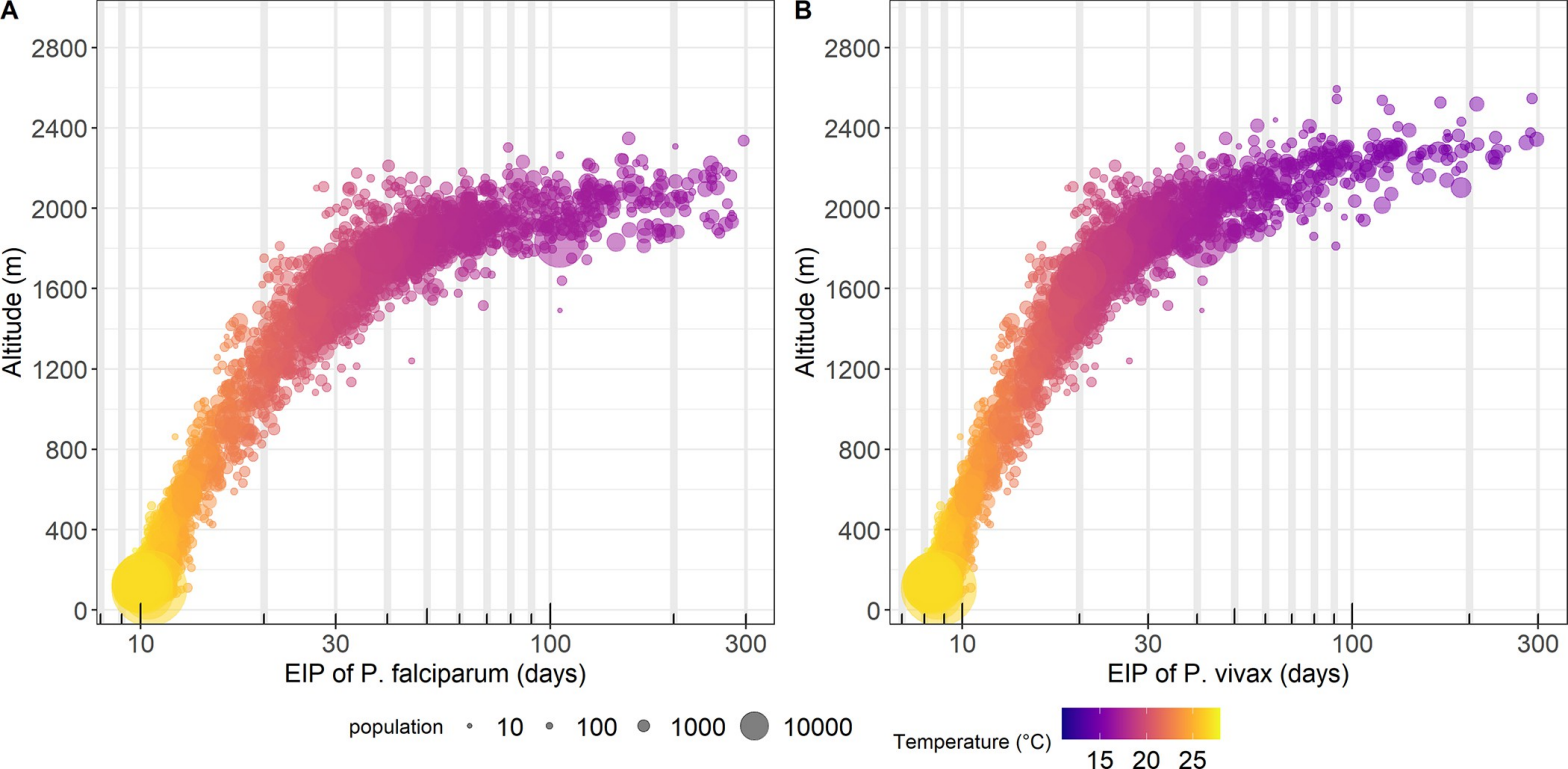
The relationship between altitude and temperature suitability for malaria in PNG. The dots represent duration of extrinsic incubation period (EIP) at the level of census wards of PNG: A) EIP for *P*. *falciparum*, and B) EIP for *P*. *vivax*.

Fig 1 shows the extrinsic incubation period, i.e. the duration of sporogony in days, versus altitude, at the level of census wards of PNG. For *P*. *falciparum*, the EIP elongates with increasing of altitude, a maximum of 16 days in areas below 1000 m (below 23°C) compared to > 40 days in areas above 1400 m (below 19°C). In contrast, EIP of *P*. *vivax* shows lower limits of temperature (and higher of altitude), i.e. a maximum of 14 days in census wards below 1000 m versus >24 days in areas above 1400 m. In areas above 2000m, EIP could be very lengthy exceeding 300 days at 2030 m and 2400 m, for *P*. *falciparum* and *P*. *vivax*, respectively. Approximately eleven percent of the PNG population lives in areas above 2000m altitude; see S4 Fig.

In a previous study, the daily survival rate of the main malaria vectors in PNG, i.e. *An*. *farauti s*.*l*. was reported in a range 0.63 to 0.72 [50]. Even for incremental changes, the duration of EIP has an exponential effect on the vectorial capacity of the mosquito [51]. Hence, local malaria transmission is hypothetically challenging to occur in higher altitudinal areas in PNG because of elongation of the EIP which could exceed the lifetime of adult mosquitoes.

### Distribution of health facilities

**Fig 2:**
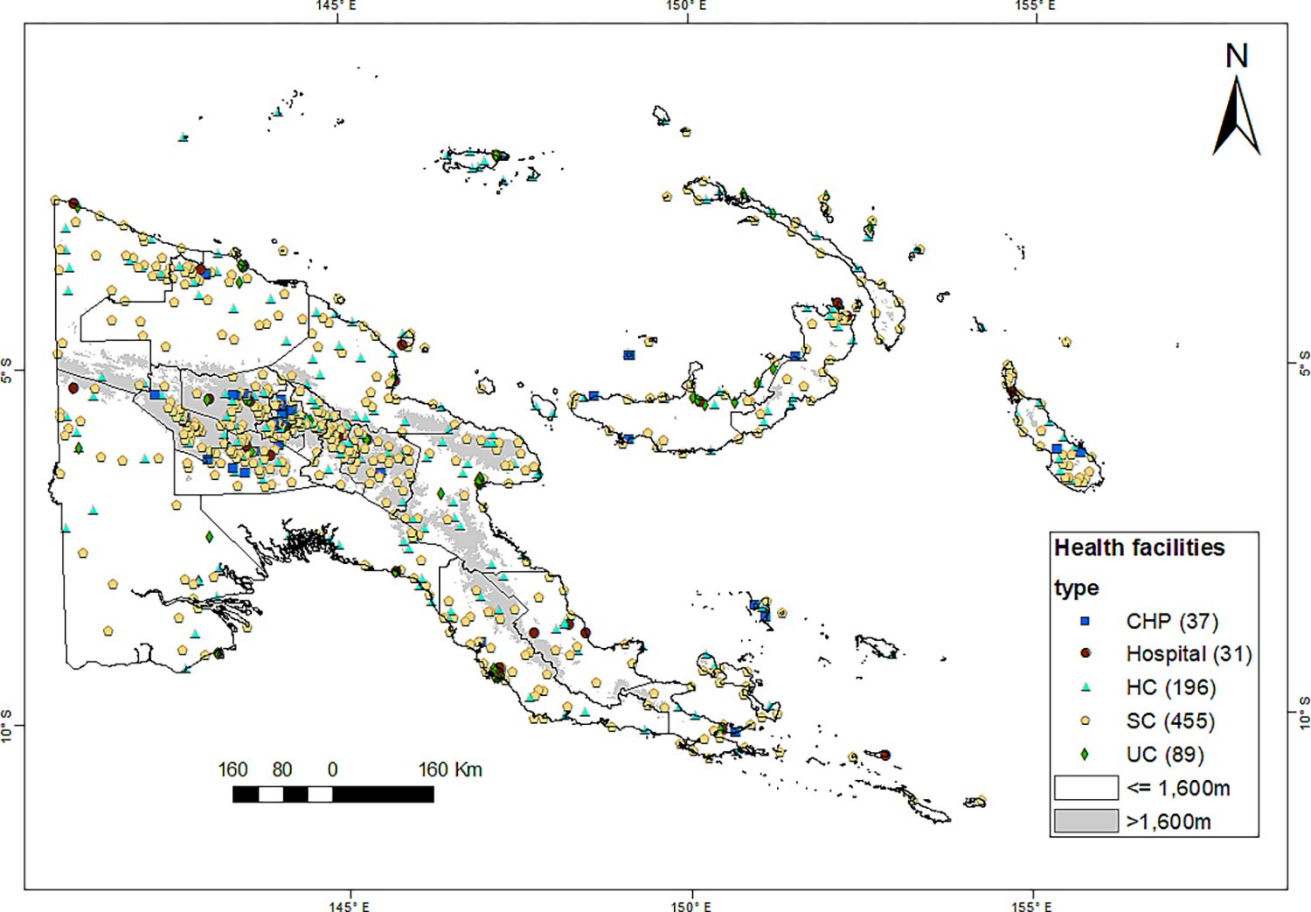
Distribution of health facilities in Papua New Guinea. Locations of 808 health facilities: HC = Health Centre; SC = Sub-Health Centre; CHP = Community Health Post; UC = Urban Clinic. Source of the map base layer: WFP-World Food Programme, 2019. Map created by the authors using a licensed ArcGIS Desktop 10.5 software from Esri (http://www.arcgis.com/).

The distribution of 808 HFs in PNG is shown in Fig 2. The mapped HFs are 31 hospitals (including ten district hospitals), 196 health centres, 455 sub-health centres, 89 urban clinics, and 37 community health posts.

Most HFs (33.8%) cluster in the Highlands Region because of the high population density. However, Morobe and Manus have the largest and smallest number of working HFs, 53 and 13, respectively. Most offshore small islands are serviced by aid posts, which are supervised by nearby health centres or sub-health centres. Due to unknown functionality status, we excluded 2672 aid posts from this analysis.

### Population distribution, altitude and access to nearest health facility

Approximately half of the population of PNG lives in coastal areas (below 800 m) and one-third in highland areas (1600–2000 m). In particular, coastal areas below 200m are home to 3.5 million people using the 2019 projection of a total of 9.6 million.

The OSM road network data for PNG (2016–2020) is twice the one used in the friction surface map from 2015, adding a total of 35,930 km of roads length, (see S2 Fig). Hence, the updated friction surface has substantially improved estimation of travel time between residential places and the HFs. The dramatic increase in mapped roads does not reflect a change in infrastructure of PNG like building of new roads, rather there is an improvement in data collection due to addition of existing (missing) roads by volunteers, including humanitarian mapping efforts [52].

**Fig 3:**
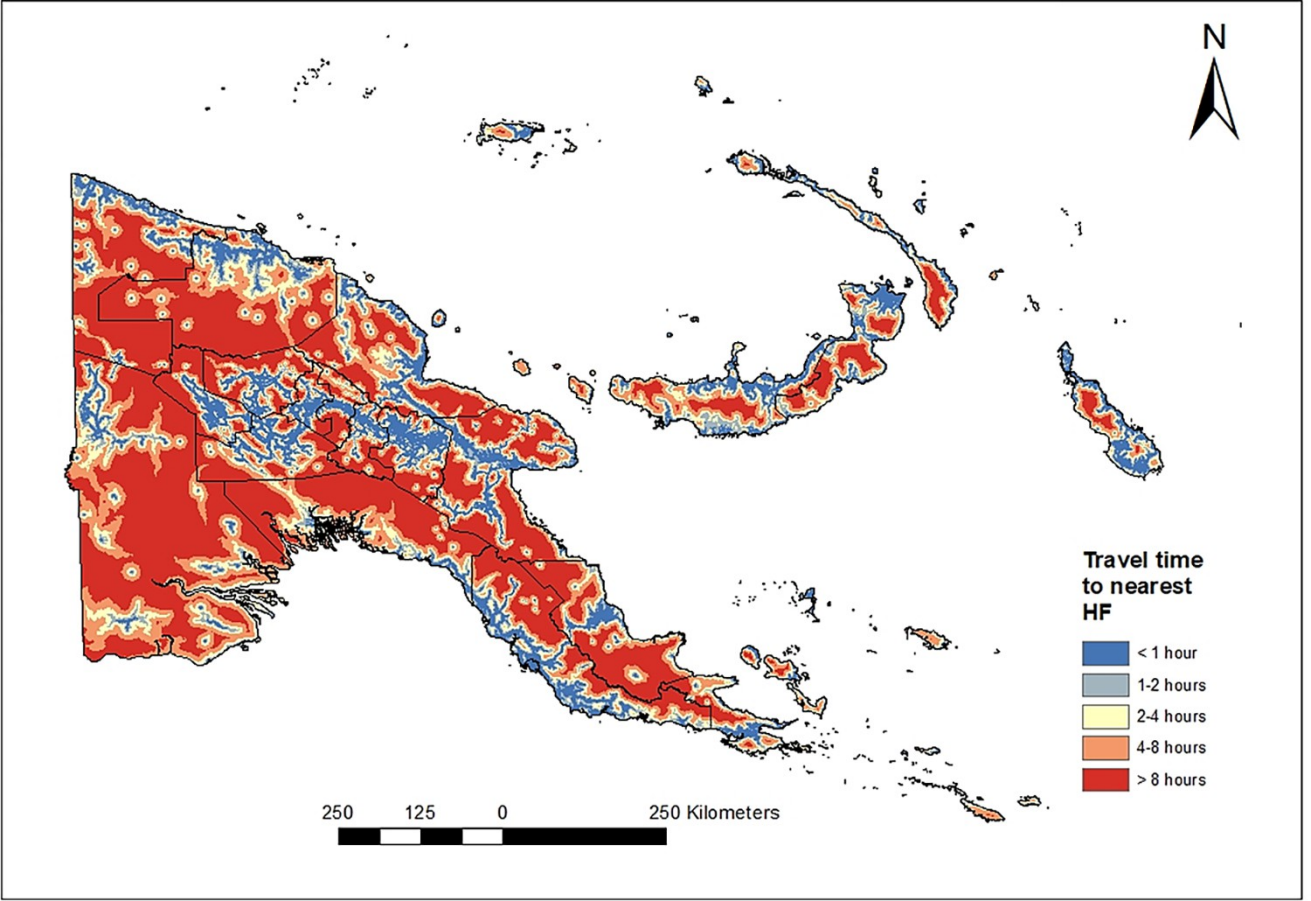
Travel time (hours) of the population from their residential places to nearest health facilities. Travel time from raster pixels (30x30 m^2^) to nearest HF was calculated using the fastest mode of transportation. Source of the map base layer: WFP-World Food Programme, 2019. Source of the map base layer: WFP-World Food Programme, 2019. Map created by the authors using a licensed ArcGIS Desktop 10.5 software from Esri (http://www.arcgis.com/).

Fig 3 shows a map of the travel time between people’s residential places and the nearest HFs. High proportions (>85%) of the populations in the National Capital District (NCD), East New Britain, New Ireland and Bougainville have easy access (i.e. travel time less than one hour) to HFs. Road networks in coastal areas and between urban centres of the Highlands reduce the travel time to less than two hours. In contrast, >30% among the population living further inland have difficult access to HFs (i.e., travel time exceeding two hours), especially in Gulf, Western, Madang, East Sepik and Sandaun provinces; see S4 Fig.

### Catchment areas of health facilities

Catchment areas of 808 HFs were delineated using the Facebook population density map for PNG. On average, the catchment population is 12,045, ranging from 812 to 149,605, at an average altitude of 850 m, ranging from four to 3033 m. While a treatment seeker needs an average of 32 minutes (range 10 to 117) to reach the closest HF, the distance from their houses averages 3170 m (100 to 7608). Characteristics of the catchment areas by province are summarised in S1 Table.

### Summary of malaria reports (2011–2019)

Table 1 summarises the NHIS malaria indicators reported by HFs during the period 2011–2019. Overall, 66,621 monthly reports were obtained; however, not all HFs had regularly reported, ranging from 705 HFs in 2013 to 783 HFs in 2019.

**Table 1:** Summary of malaria reports at health facilities, PNG (2011–2019).

Year	Health facilities	Reports received (completeness)*	Presumptive cases	Confirmed cases (positivity)**_		Total
				microscopy	mRDTs	
**2011**	727	7,947 (82%)	1,192,402	75,513 (36%)	10,161 (31%)	1,278,076
**2012**	725	7,618 (79%)	897,145	67,985 (39%)	86,395 (34%)	1,051,525
**2013**	705	6,693 (69%)	558,460	59,290 (44%)	173,489 (37%)	791,239
**2014**	733	7,252 (75%)	345,449	65,370 (50%)	202,277 (37%)	613,096
**2015**	733	7,263 (74%)	260,786	74,633 (53%)	229,561 (41%)	564,980
**2016**	735	7,223	252,129	82,481 (52%)	323,440 (43%)	658,050
**2017**	729	7,161 (74%)	224,601	70,477 (49%)	408,010 (46%)	703,088
**2018**	750	7,499 (74%)	193,697	62,973 (47%)	488,722 (48%)	745,392
**2019**	783	7,965 (82%)	178,854	39,962 (51%)	611,930 (50%)	830,746
**Overall**	**783**	**66,621 (76%)**	**4,103,523**	**598,684 (46%)**	**2,533,985 (44%)**	**7,236,192**

*The percentage of completeness calculated as the number of monthly reports received from HFs divided by the expected number of reports from total HFs (n = 808).

The total number of reported cases was 7,236,192, including 4,103,523 presumptive and 3,132,669 confirmed cases. Annual total of confirmed cases have gradually increased over time exceeding the number of presumptive cases by 2015. Availability of mRDTs (and ACTs) in HFs increased since the GFATM-supported rollout in 2011/2012, and particularly in 2018 and 2019. At the same time the mRDT positivity rose steadily from 31% in 2011 to 50% in 2019. In contrast, few positive microscopy results were reported (598,684) because of a low coverage of microscopy services.

### Estimates of malaria cases unseeking care at HFs

Proportions of patients seeking treatment at HFs by region are presented in S5 Fig. The weighted means of treatment-seeking is higher in the Islands and Southern regions compared to the Highlands and Momase, i.e. 0.543 and 0.532 vs. 0.441 and 0.446, respectively. Table 2 displays estimates of malaria patients unseeking care among general population and children under five years by region. Overall, we estimated a total of 2,466,279 malaria cases including 627,447 children under five years that did not sought treatment and not reported to the health facilities of PNG between 2011 and 2019. The highest and lowest proportions of these cases remained at home without treatment were in Momase and Highlands regions, 52.5% and 4.4%, respectively.

**Table 2:** Estimates of malaria cases unseeking care by region in PNG (2011–2019).

year	Highlands Region		Islands Region		Momase Region		Southern Region	
	All	Children U5 years	All	Children U5 years	All	Children U5 years	All	Children U5 years
2011	29,012	8,013	76,464	25,890	153,618	47,086	54,263	18,389
2012	16,534	3,498	81,699	26,030	107,111	31,634	45,769	14,017
2013	9,683	2,178	71,989	24,290	96,715	26,272	45,463	12,185
2014	10,424	1,434	41,684	14,352	126,324	32,671	25,533	6,324
2015	7,823	1,127	48,170	14,442	117,553	29,522	32,791	7,384
2016	7,228	831	67,430	20,446	131,205	28,687	52,809	11,423
2017	8,311	760	73,778	21,846	179,286	38,875	39,637	8,598
2018	9,786	910	76,868	20,319	179,737	38,116	64,425	12,193
2019	11,628	867	96,144	24,890	204,095	40,000	65,290	11,946
overall	110,430	19,618	634,226	192,505	1,295,644	312,863	425,980	102,460

### Malaria incidence and altitude

**Fig 4:**
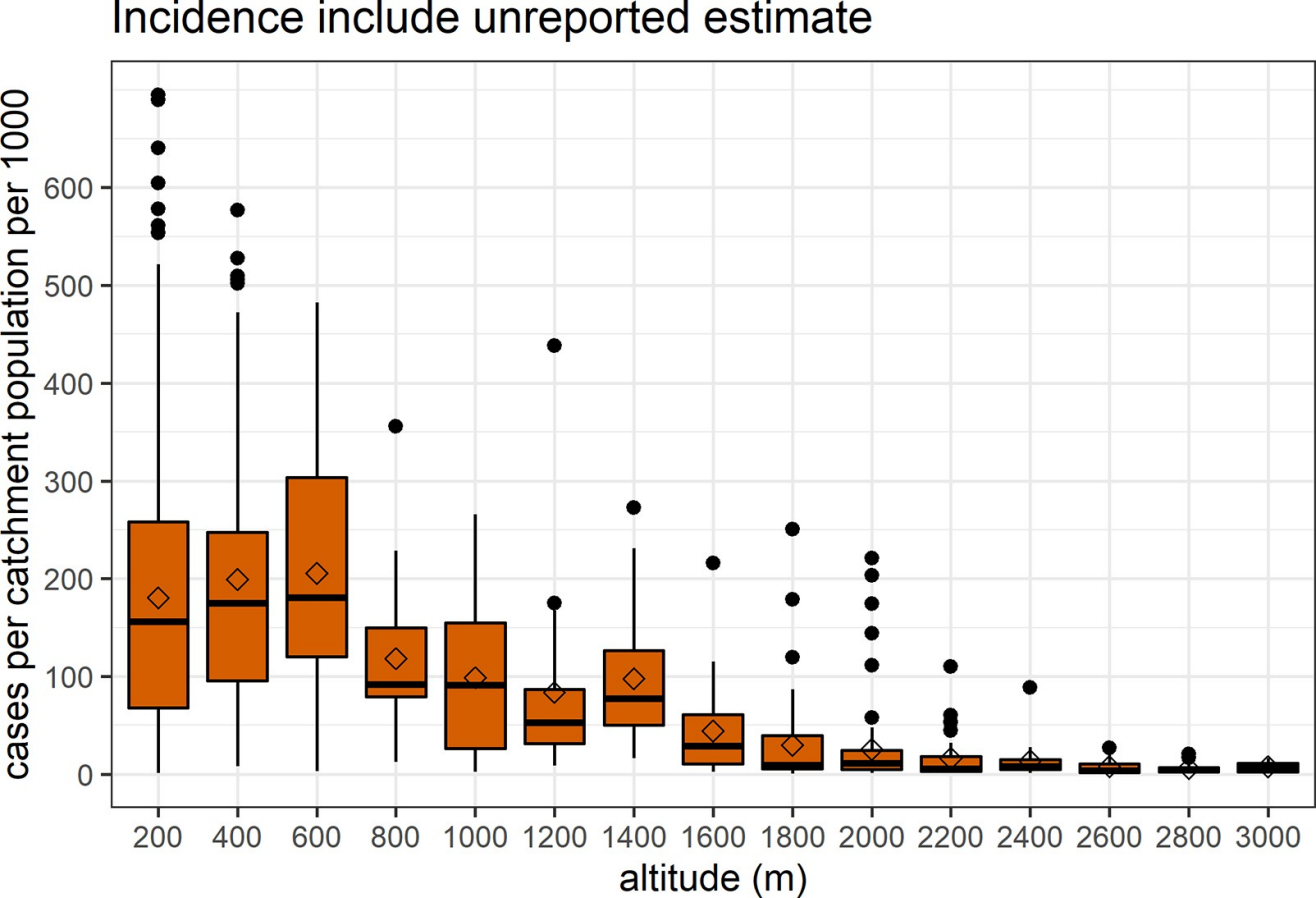
The average annual malaria incidence (per 1000 population) in catchment areas of health facilities, PNG (2011–2019). The average altitude (meters) and estimated population within two hours travel distance are used.

Overall, the average annual incidence (2011–2019) at catchment areas of HFs was 125.2 [95% CI 115.5, 134.8] cases per 1000 population. The incidence rates significantly decrease from 186.3 [95% CI 173.1, 199.4] in areas up to 600 m, to 98.8 [95% CI 11.1, 77] at (800–1400) m, to 24.1 [95% CI19.4, 28.8] in catchments above 1400 m (adjusted R^2^ = 0.29, p<0.001), see Fig 4. In areas above the two altitudinal thresholds 600 m and 1400 m, the average annual temperature drops below 22°C and 17°C, respectively.

**Fig 5:**
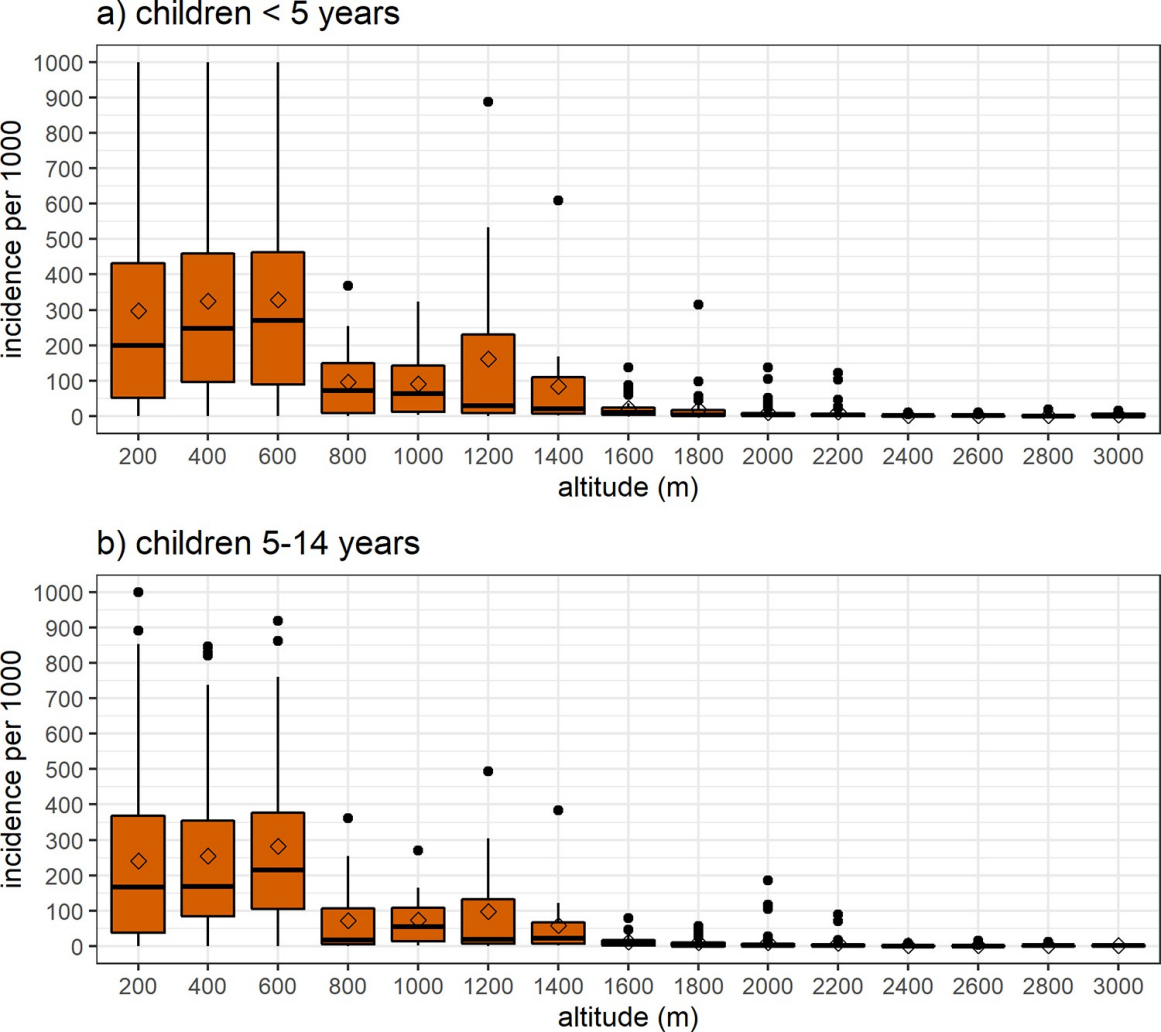
Average annual malaria incidence in catchment areas of health facilities among age groups < 15 years, PNG (2011–2019). Incidence rates per 1000 among: a) children under five years old (U5), c) adolescents (5–14) years old.

Fig 5 confirms previous findings on incidence and altitude among age groups < 15 years, who are expected to be less mobile and could reflect local malaria transmission in their catchment areas. Hence, annual incidence among children under five years significantly declined at areas above the 600 m and 1400 m: from 305 [95% CI 277.1, 332.8] to 109.9 [95% CI 68.1, 151.7] and 12.3 [95% CI 8.7, 15.8] cases per 1000, respectively (Fig 5A). Similarly, in the adolescents aged 5–14 years, the rates decreased from 245.8 [95% CI 223.6, 268] to 76.5 [95% CI 49.5, 103.6] and to 8.3 [95% CI 5.9, 10.6], respectively (Fig 5B).

### Persistence and inter-annual variability of incidence (2011–2019)

**Fig 6:**
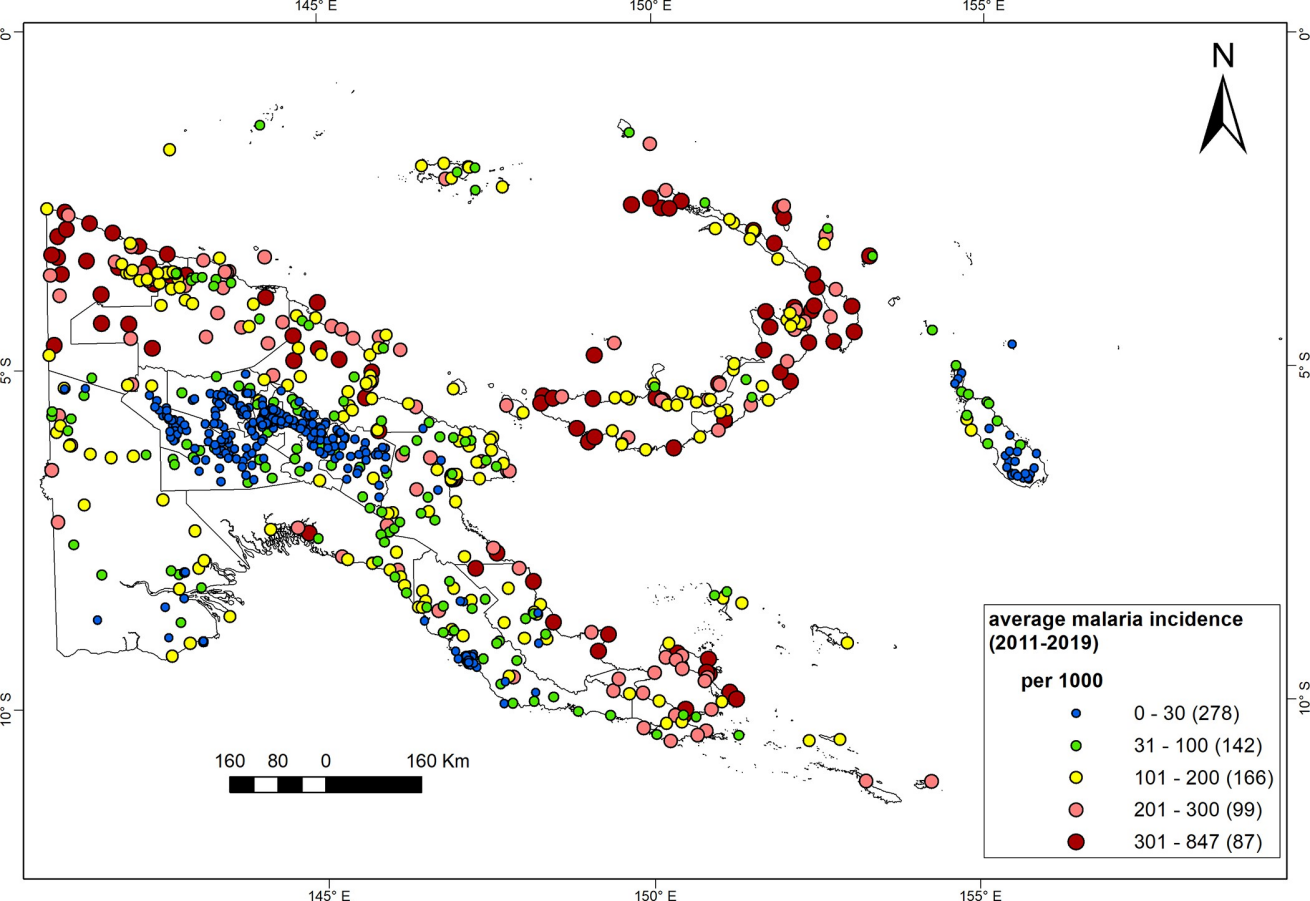
The average annual malaria incidence per 1000 in catchment areas of 772 health facilities, PNG (2011–2019). Annual incidences were calculated as sum of adjusted presumptive and confirmed (using light microscopy or mRDTs) cases and estimates of patients unseeking care, among the population in the catchment area of the health facility. Source of the map base layer: WFP-World Food Programme, 2019. Map created by the authors using a licensed ArcGIS Desktop 10.5 software from Esri (http://www.arcgis.com/).

Fig 6 shows the average annual incidence (2011–2019) in 772 HFs with continuous reporting—out of 808 geocoded HFs—across PNG. Malaria incidence is very high (>300 cases per 1000) to high (> 200 to 300 cases per 1000) in 24% of the HFs, mainly in Sandaun, Madang, Milne Bay, New Britain and New Ireland. In contrast, 54% of the catchment areas, mainly in the Highlands, Bougainville and NCD, have a low (31 to 100 cases per 1000) to very low incidence (0 to 30 cases per 1000).

**Fig 7:**
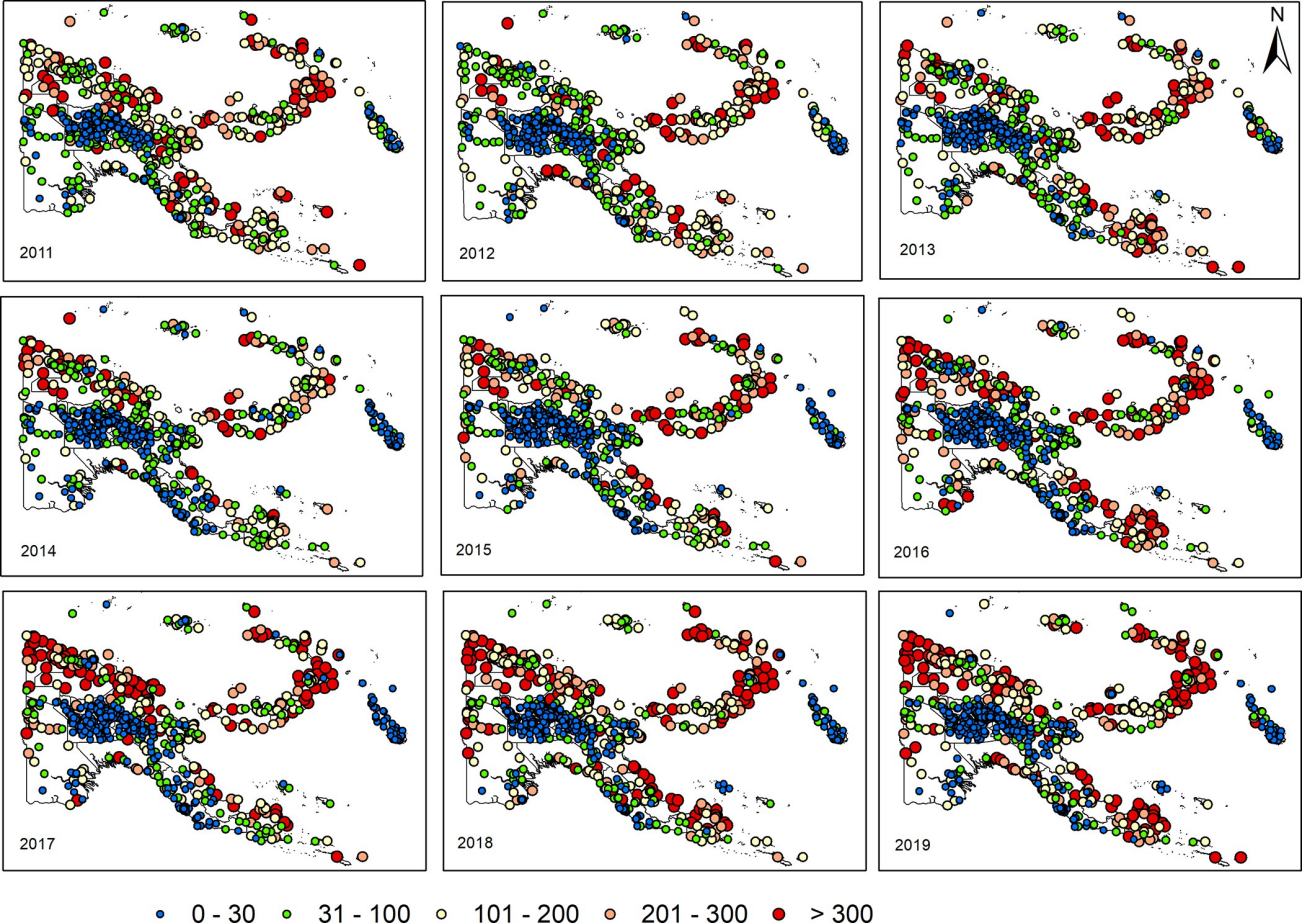
Yearly maps of annual malaria incidence in catchment areas of 806 health facilities, PNG (2011–2019). Five categories of incidence per 1000: > 300 (red), 300–200 (pink), 200–100 (yellow), 100–30 (green), and <30 (blue). Incidence was calculated as annual sum of adjusted presumptive and confirmed (positive microscopy or mRDTs) malaria cases and estimates of patients unseeking care, among the catchment’s population. Source of the map base layer: WFP-World Food Programme, 2019. Map created by the authors using a licensed ArcGIS Desktop 10.5 software from Esri (http://www.arcgis.com/).

Yearly maps of annual incidence show a change of malaria risk across PNG from 2011 to 2019, see Fig 7. Apparently, increasing patterns of malaria incidence are observed mainly in Momase and Southern Regions in the last three years (2017–2019) compared to the years (2013–2015). The coastal areas in Sandaun, Milne Bay, New Britain, and New Ireland are among the provinces with catchment areas demonstrating high inter-annual variability. On the contrary, the low incidence settings have largely remained unchanged in Highlands Region. Yearly average incidences by province are shown in S6 Fig.

### Catchment areas with few malaria cases among children and adolescents below 15 years

**Fig 8:**
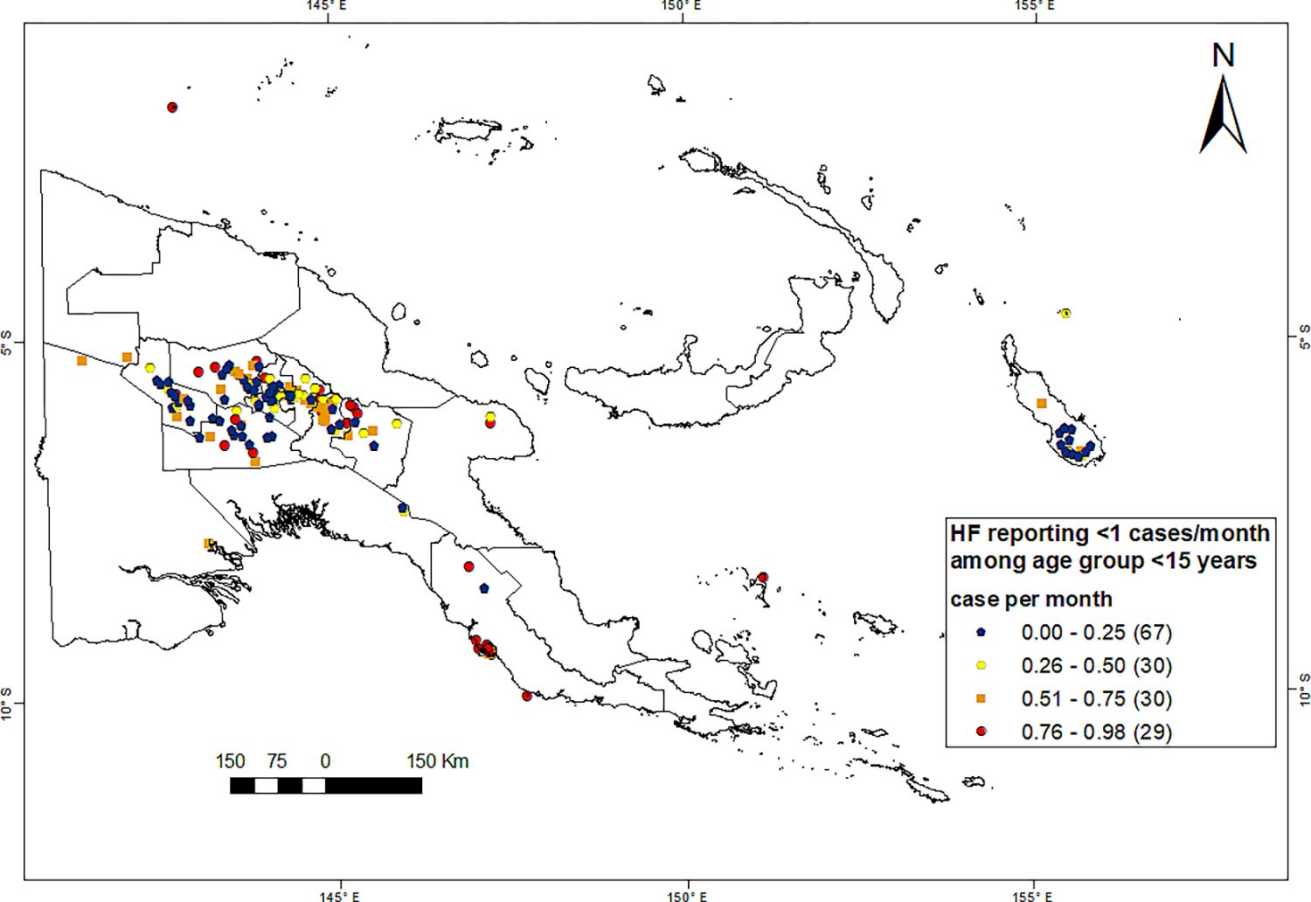
Health facilities (HFs) reporting few malaria cases in children <15 years (2011–2019). The average number of malaria cases in children and adolescents registered per month per HF is less than one. Source of the map base layer: WFP-World Food Programme, 2019. Map created by the authors using a licensed ArcGIS Desktop 10.5 software from Esri (http://www.arcgis.com/).

Overall, 26% of the PNG population (i.e., 2.3 million) live in catchment areas of HFs that reported few cases in children and adolescents. In 156 HFs, the average monthly number of malaria cases among the population under 15 years of age amounted to less than one case (2011–2019). These HFs are mainly located in the Highlands, Bougainville and NCD, see Fig 8. The positivity rate microscopy slides and mRDT) averaged 9% among the children <15 years but was higher in the Highlands (>1600m) than in coastal areas (see Table 3).

**Table 3:** Health facilities with few reported malaria cases in children < 15 years (on average, less than one case per monthly report) by altitude, PNG (2011–2019).

Altitude (m)	No. HFs	U15 cases^1^	Test positivity^2^	Total Population	Total LLINs issued^3^
**≤ 400**	28	959 (32.9%)	5.8% (1.6, 14.6)	261,563	105,248
**> 400–800**	3	79 (22.8%)	5.1% (1.1, 8.2)	21,233	4,999
**> 800–1200**	4	147 (37.1%)	6.6% (3.1, 12.8)	41,793	7,486
**> 1200–1600**	10	173 (28.7%)	11.8% (0, 28.6)	112,782	40,076
**> 1600–2000**	66	1,141 (20.7%)	8.6% (0, 31.2)	1,076,492	464,496
**> 2000–2400**	22	531 (19.4%)	10.2% (0.3, 30.6)	376,782	170,702
**> 2400–2800**	20	281 (14.5%)	13% (0, 42.1)	385,575	147,478
**> 2800**	3	55 (18.7%)	11% (4.9, 19.9)	193,69	12,186
**All**	**156**	**3,366 (22.9%)**	**9% (0, 42.1)**	**2,295,589**	**952,671**

^1^ Total confirmed cases of individuals under 15 years (U15), proportion relative to confirmed cases among the general population are in parenthesis.

^2^ Average positivity rates among tested U15, minimum-maximum rates are in brackets.

^3^ Total LLINs were distributed in the catchments areas (2011–2019).

### Strata of malaria incidence using EBK models

#### Cross-validation of EBK models

Diagnostics of cross-validation in ArcGIS show that chosen parameters resulted in good fitting models using incidence both for the general population and children < 15 years (see S2 Table).

RMSSE values using the incidence of the general population and children < 15 years indicate reasonable variability in the predictions, 1.01 and 1.04, respectively. The values of RMSE vs ASE are close together, 87.4 vs 92.2 and 201.2 vs 204.8, general population and age group < 15 years, respectively. However, the mean prediction errors were -0.32 and 0.3, in the general population and children < 15 years (see S7 Fig). The correlation coefficient at locations of HFs between observed incidence rates and EBK predicted values shows a strong positive relationship, r(770) = 0.93 and 0.89, p < .001, respectively.

#### Strata of malaria risk

**Fig 9:**
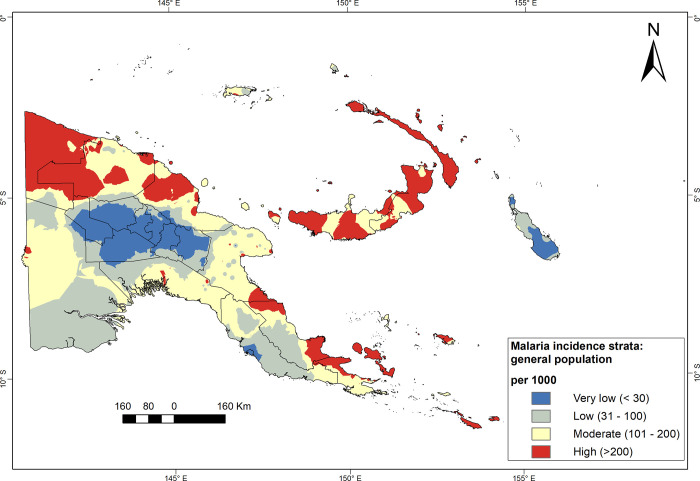
Malaria risk strata using the average annual incidence of cases among the general population, PNG, 2011–2019. Four strata interpolated using empirical Bayesian kriging at catchments of HFs: very low (<30), low (30–100), moderate (100–200), and high (>200 cases per 1000). Source of the map base layer: WFP-World Food Programme, 2019. Map created by the authors using a licensed ArcGIS Desktop 10.5 software from Esri (http://www.arcgis.com/).

**Fig 10:**
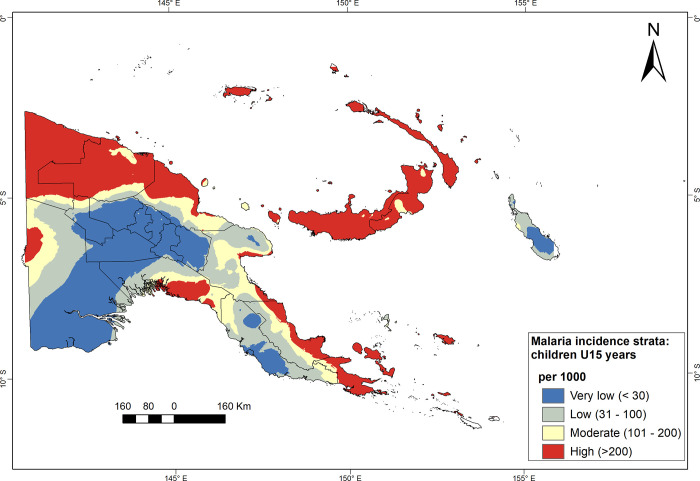
Malaria risk strata using the average annual incidence of cases among children <15 years, PNG, 2011–2019. Four strata interpolated using empirical Bayesian kriging at catchments of HFs: very low (≤30), low (30–100), moderate (100–200), and high (>200 cases per 1000). Source of the map base layer: WFP-World Food Programme, 2019. Map created by the authors using a licensed ArcGIS Desktop 10.5 software from Esri (http://www.arcgis.com/).

Malaria risk strata using average annual incidence for 2011–2019 are presented for the general population (Fig 9), and children under 15 years (Fig 10). Interpolations of incidence rates across catchment areas of HFs resulted in similar spatial patterns but different in magnitude regardless of used indicator (i.e. among the general population versus the group of children and adolescents under 15 years).

Nevertheless, modelled areas with a high malaria risk, mainly in the Islands, Momase, and Milne Bay province, were more prominent using incidence among the general population than the subpopulation under 15 years. In contrast, the risk of malaria is very low or low in the Highlands and the Southern Region (NCD, Central and Western provinces) and Bougainville using incidence among the subpopulation under 15 years. Besides, moderate risk strata predominate throughout the mainland provinces, especially Morobe, Western and Oro provinces.

### Population by malaria risk strata

As of 2019, 35.7% of the PNG population (ca. 3.33 million) live in areas at high or moderate risk of malaria (Table 4). In five provinces, relatively large proportions of the population (> 50%) reside in high-risk areas: New Ireland, East and West New Britain, Sandaun and Milne Bay. In contrast, very low-risk strata, which are 52.2% of the country’s population (ca. 4.88 million), cluster in the Highlands Region and Bougainville.

**Table 4:** Population distribution of PNG by province according to malaria incidence strata, 2019.

Province	HFs^(a)^	Population 2019^(b)^	% population living at risk strata^(c)^			
			Very low	Low	Moderate	High
**Bougainville**	35	293,911	52.3%	46.4%	1.2%	0.2%
**East New Britain**	32	304,971	0.0%	0.0%	33.8%	66.2%
**Manus**	13	52,291	0.0%	28.9%	65.2%	5.9%
**New Ireland**	32	205,671	0.0%	2.2%	8.2%	89.7%
**West New Britain**	36	296,584	0.0%	0.0%	47.1%	52.9%
**Central**	41	284,664	21.3%	58.0%	20.7%	0.0%
**Gulf**	21	124,111	1.0%	13.0%	79.9%	6.1%
**Milne Bay**	43	319,032	0.0%	0.0%	45.2%	54.8%
**National Capital District**	25	364,895	100.0%	0.0%	0.0%	0.0%
**Oro**	19	216,629	0.0%	51.7%	32.8%	15.5%
**Western**	42	191,299	0.1%	64.7%	34.6%	0.6%
**Chimbu**	35	631,307	94.8%	5.2%	0.0%	0.0%
**Eastern Highlands**	37	739,063	92.4%	7.4%	0.2%	0.0%
**Enga**	37	706,130	94.8%	5.1%	0.1%	0.0%
**Hela**	32	691,462	93.4%	6.1%	0.5%	0.0%
**Jiwaka**	28	645,409	89.9%	10.1%	0.0%	0.0%
**Southern Highlands**	41	714,659	96.9%	3.1%	0.0%	0.0%
**Western Highlands**	41	473,708	90.2%	9.8%	0.0%	0.0%
**East Sepik**	45	504,438	0.0%	9.5%	55.1%	35.3%
**Madang**	48	608,359	0.1%	7.4%	62.5%	30.0%
**Morobe**	53	715,789	0.3%	20.8%	63.4%	15.5%
**Sandaun**	36	260,543	0.0%	6.3%	16.1%	77.6%
**PNG (all provinces)**	**772**	9,344,925	52.2%	12.1%	20.3%	15.4%

(a) Number of health facilities functioning and reporting on malaria to NHIS, NDOH.

(b) Projected population in 2020 by province based on a district-specific growth rate, NSO, PNG.

(c) Breaks of risk strata based on the interpolated surface of incidence per 1000: very low (<30), low (30–100), moderate (100–200), and high (>200).

## Discussion

The main contributions of this stratification work are three-fold. First, we used routinely collected health information system data to map incidence and stratify the risk of malaria in PNG at a microscale of catchment areas of HFs. Second, we determined altitudinal thresholds that influence malaria risk and examined the effects on different population groups. Third, we delineated the catchment areas of HFs and estimated the population at risk by strata using geostatistical modelling methods.

Previous studies had documented the negative relationship between malaria and altitude in PNG [18, 20, 21, 53–55]. Our work quantified the relationship between altitude and malaria incidence at a high resolution, i.e. catchment areas of health facilities. We found two altitudinal thresholds at 600 m and 1400 m where the average temperature drops below 22 and 17°C, respectively, and malaria incidence declines substantially. In the context of global changes in average temperature, a warmer climate could increase the risk of malaria epidemics, especially among nonimmune populations in highland areas, unless the importation of infections is prevented. Prior research showed parallel increases in annual temperature (0.33°C per year) and malaria incidence (3.15 per 1000) in the Highlands from 1996–2007 [56].

The malaria incidence strata presented here are coherent with the results of the previous five malaria indicator surveys (MIS) conducted in 2008/09, 2010/11, 2013/14, 2016/17 and 2019/20 [12, 44, 45, 57]. Incidence maps confirm a low malaria risk across the central mountain range in the Highlands, Bougainville, and NCD. In contrast, Momase, Islands and Milne Bay provinces exhibit patches of high risk. In addition, strata of moderate risk predominate throughout the coastal areas on the mainland of PNG. In the latest malaria indicator surveys (2019/20), the lowest prevalence was found in the Highlands (0.03%), while Momase Region (4.1%) had the highest prevalence [45]. Provinces with the highest prevalence included Sandaun (10.6%), East Sepik (8.6%), Oro (3.7%), East New Britain (2.6%), Madang (2.5%), and Milne Bay (2.2%) [45].

We also identified 156 HFs that report on average less than one malaria cases per month among children and adolescents < 15 years. These were located mainly in the Highlands Region and in Bougainville. Even though almost one million LLINs were distributed in the catchment areas of HF reporting low case numbers between 2010–2019, in the Highlands and Bougainville, only 45.5% of people with access to an LLIN actually used a net [45]. Low incidence rates in spite of low rates of LLINs use suggest that certain areas in the Highlands are indeed less receptive to malaria.

The stratification of malaria risk across PNG using routine incidence data applied at a microscale level of HF catchment areas provides for the first time since the 1970s [17] a comprehensive overview of the differential malaria risk faced by the people of PNG. Nevertheless, a limitation of this work is the limitation of the analysis to the period 2011–2019. A more comprehensive work could include additional historical data as well as individual patient-level malaria register data including the village of residence of patients, ideally complemented by parasite and mosquito surveys.

The purpose of this stratification is to contribute to better sub-national tailoring and targeting of malaria control interventions across PNG. The NMCP should prioritise catchment areas with high and moderate risk in allocating interventions aimed at reducing transmission and morbidity. For example, to increase the replacement rate of effective LLINs in transmission hotspots and prevent the shortage of ACTs to alleviate the malaria burden. In contrast, there is a need for an effective surveillance-response system in low-risk areas to avoid the risk of severe epidemics. The surveillance system may complement (or, in some cases, replace) blanket coverage with other interventions if local transmission is unlikely. Since 2014, an electronic national health information system (eNHIS) has been rolled out in PNG [58]. The cost of strengthening the eNHIS or maintaining sentinel surveillance sites may be more cost-effective than providing high coverage with malaria commodities in areas with low incidence of primarily imported cases.

We found that catchments in the coastal provinces, mainly Sandaun, Milne Bay, New Ireland, and the two provinces of New Britain, exhibit high inter-annual variability. The reasons for these fluctuations in these holo-endemic areas are likely the effect of the interventions and the subsequent impact of reduced coverage/effectiveness [6]. Malaria testing and treatment are provided free of charge in health facilities in PNG, potentially decreasing the obstacles for sick people to visit public health facilities. Although the size of the catchment area may be influenced by the capacity and change over the years, we are unable to consider these factors due to lack of empirical data.

The case incidence reported at HF is a function of treatment seeking and reporting completeness. The MIS 2019/20 suggests that only 57% of febrile patients seek treatment in PNG [45]. To better evaluate the community-level disease burden based on facility -reported data we estimated totals of patients unseeking care at catchment areas of HFs. However, adjusting case counts for non-reporting requires many assumptions for which we lack the supporting evidence. Instead we excluded facilities that report only rarely and are hence unlikely to see a large enough number of patients to change the average incidence estimate.

Stockouts of mRDTs and antimalarials at health facilities occur across PNG (Bella-Sil B., RAM, personal communication). In addition, recent work has reported poor quality of LLINs distributed in PNG after 2013 based on bio-efficacy assay against the local malaria vector [59]. Hence, a shortage of malaria commodities (i.e., ACT and mRDTs) at coastal catchments could result in severe epidemics and need better resource mobilisation. Since 2018, distribution of malaria commodities has been strengthened by quarterly visits of accessible health facilities (almost >80% facilities) by regional malaria coordinators. However, a better resource mobilization is needed to reach the 20% of hard to reach facilities (RAM, personal communication, 2022).

Analysis of seasonal patterns of malaria incidence was beyond the scope of this work. Previous studies showed a limited seasonality in the lowlands of the Momase Region [5, 15, 60] but a pronounced seasonal cycle in the Islands and Southern Regions, including NCD [17, 61]. Besides, severe malaria epidemics were reported in the Highlands between April and July at altitudes 1300–1600 m, i.e. during the transition from rainy to dry season [3, 16, 22, 54, 62]. However, these epidemics may relate to seasonal migrations for agriculture purposes during the rainy season towards the beginning of the dry season.

We did not investigate the effect of population mobility on malaria risk. In PNG, there is a continuous movement between the Highlands and coastal provinces for trade (e.g., betel nuts and vegetables) and other reasons. Also, Highlanders frequent intermountain valleys for subsistence farming. In addition, there are thousands of migrant workers in mines companies (e.g., Newcrest Mining in Lihir and Ok Tedi in the Western Province) and developmental projects (e.g., PNG LNG), who transit through malarious areas [63, 64]. Hence, the socio-economic factors that govern population movements between different risk areas are important for the stratification of malaria risk. In addition, spatial weights of socio-economic ties of inhabitants in catchment areas with other ones should be considered in malaria stratification.

Previous studies showed a difference in distribution and vectorial capacity between malaria mosquitoes in PNG that have implications for malaria transmission and vector control. Temporal and spatial heterogeneities in species composition and ecology were reported even between nearby villages [14, 15, 65]. Up-to-date information on vector distribution, biting behaviour, and vectorial capacity, should be considered in the tailoring of targeted vector control.

This work did not consider a differentiation between *Plasmodium* species. Unfortunately, the data available from the NHIS severely limits our ability to take species into consideration in our work. Here is why: over the course of the studied period, there was a transition from diagnosing malaria presumptively and using microscopy (though functioning microscopy services were available only in larger facilities) to using RDTs. The RDTs used in PNG since their introduction (scale-up happened in 2012) have been HRP2/pan (pLDH) combo tests. They detect Pf-specific HRP2 (1^st^ test line) and pLDH expressed by all four *Plasmodium* species. The challenge with the result displayed by the RDT is that 1) two test lines may be due to a *Pf* mono-infection or a mixed infection of *Pf* and another species, and 2) a single pLDH test line may be due to any non-*Pf* species. Hence, the RDTs as used in PNG do not allow a clear distinction between parasite species for the purpose of a species-specific analysis. In the pre-RDT time, the same applies for all the cases diagnosed presumptively. We therefore suggest that an analysis of the species-specific malaria “risk” should be derived from methodologies that allow for a clear species diagnosis such as prevalence surveys or sentinel surveillance in selected sites.

In addition, the quality of differentiation between *Plasmodium* species is questionable in peripheral facilities. Previous studies showed a change in species composition in PNG following the "unaccomplished" control and eradication efforts in the 1970s [17, 66]. Hence, *P*. *falciparum* has prevailed over the country [5, 9, 60], while *P*. *vivax* remained dominant only in low-risk areas at the Highlands [54]. Relative increases of infections by *P*. *vivax* during epidemic years were observed in two sentinel sites in New Ireland and Madang [6].

Cross-validation of the EBK models showed satisfactory performance in their predictions verified by the five error-related statistics. EBK are among geostatistical modelling tools has the advantage of being more accurate for small datasets. In contrast, large datasets increase the computing time needed to produce raster maps of EBK. We tried other geostatistical methods—such as Inverse Distance weighting, simple kriging and cokriging—but we found EBK is more suitable to model incidence data with minimal prediction error. Nevertheless, there is a need to investigate the role of altitude and other predictors using Bayesian regression kriging methods [67].

The incidence strata identified in this work provide no absolute risk of malaria infection at individual level, rather a relative risk to characterise the heterogeneity across PNG with aim to facilitate targeting interventions approaches. Although WHO provides cut-offs guidance [48], the strata may have to be adapted for each country, as done in this report, to accommodate local levels of heterogeneity and ensure sufficient discrimination between different areas. A similar approach to the one applied in this work has previously been used elsewhere, for example, in Tanzania [24]. Modelling tools have been used to stratify malaria and improve national malaria programs’ decision-making [24, 26, 68–71]. Both geostatistical and dynamic modelling methods could assist decision-making on malaria control by examining different allocation scenarios of interventions at operational units.

## Conclusions and recommendations

Altitude is among the risk factors to stratify malaria in PNG. Stratification maps show clustering of very low to low-risk strata in provinces of Highlands, NCD and Bougainville. In contrast, modelled high and moderate risk patches are mainly in Momase, Islands, and Southern Regions. We estimated 35.7% of the PNG population lives in areas at high or medium risk of malaria (i.e., in catchment areas with >100 cases per 1’000 population per year). However, the risk of malaria is highly variable in low-lying catchment areas and needs further research into drivers of the local epidemiology to identify suitable intervention packages.

There is a need to support the PNG national malaria control programme in the tailoring and targeting malaria control interventions at a sub-national scale using disease modelling tools and building the country’s capacity for implementing a data-driven control approach.

## Supporting information

S1 Fig Administrative and elevation maps of Papua New Guinea. a) Administrative regions and provinces of Papua New Guinea. b) The elevation map of Papua New Guinea. The map shows altitude at a 90m resolution with a height accuracy of one meter. PNG has a central mountain range ranging northeast to southwest along the main island of the country. Data source: Global 3D elevation model TanDEM-X.
(DOCX)

S2 Fig Cumulative length of roads added to OSM data for PNG (2007–2020). (TIF)

S3 Fig Maps of annual means and seasonal changes of temperature and precipitation in PNG (1970–2000). (DOCX)

S4 Fig Population distribution maps of PNG. (DOCX)

S5 Fig Proportions of patients seeking care at HFs by region in PNG using MIS datasets (2013–2020). (TIF)

S6 Fig Average malaria incidence by province among general population (2011–2019), PNG. (TIF)

S7 Fig Cross-validation of empirical Bayesian kriging (EBK) models. (TIF)

S1 Text Statistics of cross-validation of EBK models. (DOCX)

S1 Table Characteristics of health facilities (HFs): Averages of population, altitude, travel time and travel distance. Min-Max values are in parentheses.
(DOCX)

S2 Table Parameters used in EBK models for stratification of malaria incidence in general population and children under 15 years. (DOCX)
