# Supplementary material for: Stratification of malaria incidence in Papua New Guinea (2011–2019): Contribution towards a sub-national control policy
Source: PLOS Glob Public Health. 2022 Nov 21;2(11):e0000747. doi: 10.1371/journal.pgph.0000747 (PMC10022348; doi:10.1371/journal.pgph.0000747)
Supplement: S1 Fig — a) Administrative regions and provinces of Papua New Guinea. b) The elevation map of Papua New Guinea. The map shows altitude at a 90m resolution with a height accuracy of one meter. PNG has a central mountain range ranging northeast to southwest along the main island of the country. Data source: Global 3D elevation model TanDEM-X. (DOCX) [file pgph.0000747.s001.docx]

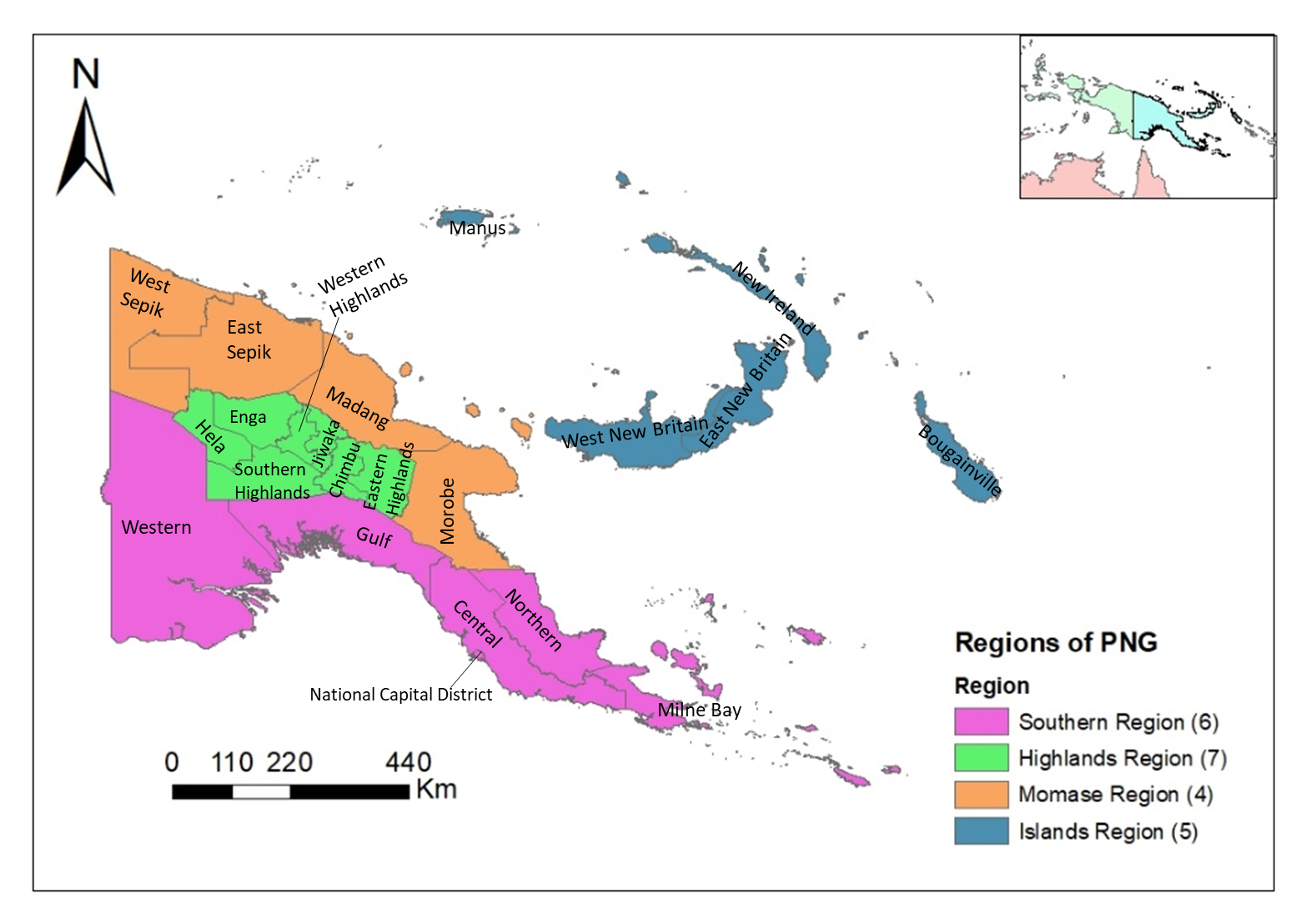


**S1 Fig. a)** Administrative regions and provinces of Papua New Guinea.


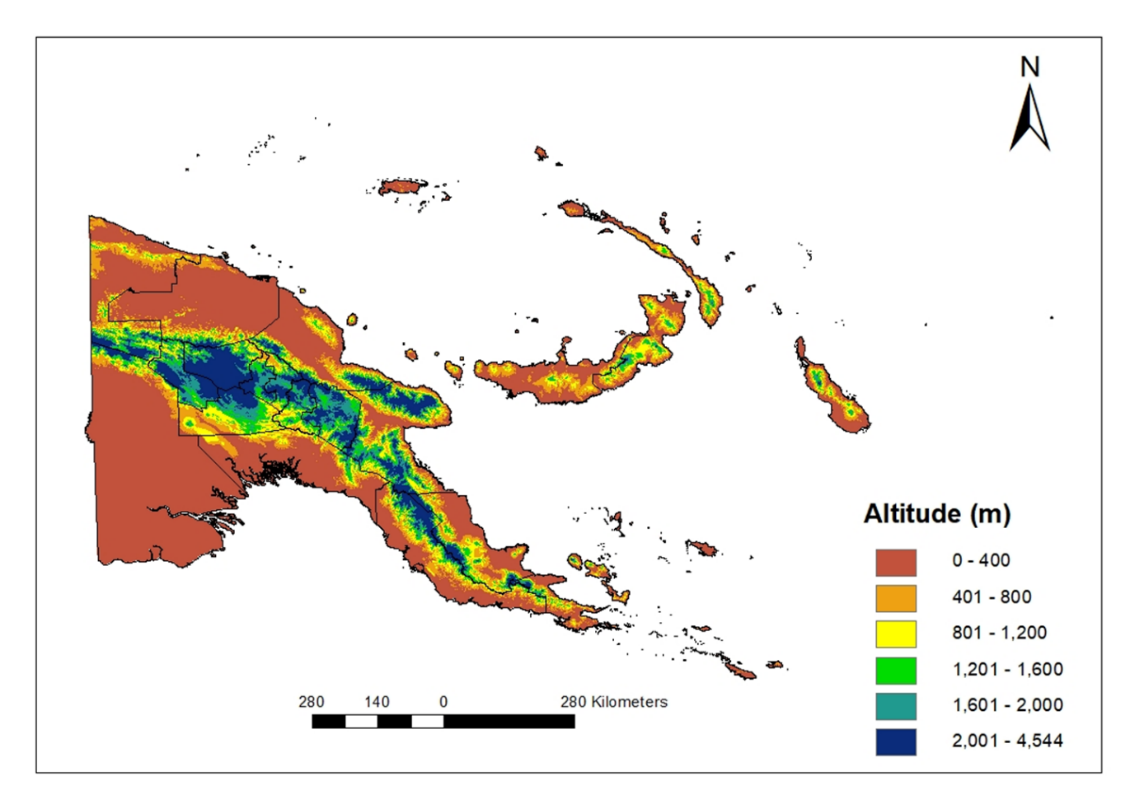


**S1 Fig. b)** The elevation map of Papua New Guinea. The map shows altitude at a 90m resolution with a height accuracy of one meter. PNG has a central mountain range ranging northeast to southwest along the main island of the country. Data source: Global 3D elevation model TanDEM-X.
