# Supplementary material for: Stratification of malaria incidence in Papua New Guinea (2011–2019): Contribution towards a sub-national control policy
Source: PLOS Glob Public Health. 2022 Nov 21;2(11):e0000747. doi: 10.1371/journal.pgph.0000747 (PMC10022348; doi:10.1371/journal.pgph.0000747)
Supplement: S3 Fig — (DOCX) [file pgph.0000747.s003.docx]

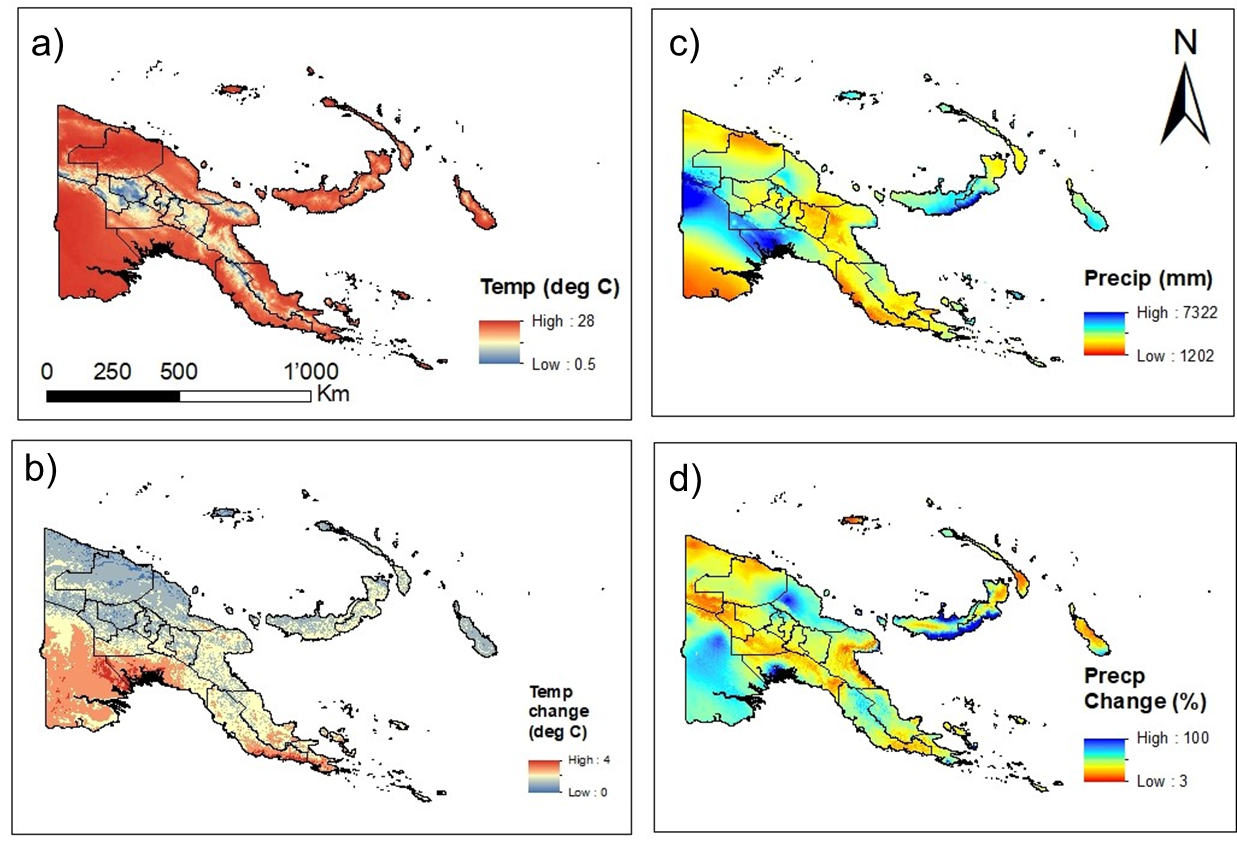


**S3 Fig.** Annual means and seasonal changes of temperature and precipitation in PNG (1970-2000). a) Annual average temperature (℃), b) seasonal change of temperature (warmest-coldest month), c) annual average precipitation (mm), d) seasonal change of precipitation (wettest-driest month).
