# Supplementary material for: Stratification of malaria incidence in Papua New Guinea (2011–2019): Contribution towards a sub-national control policy
Source: PLOS Glob Public Health. 2022 Nov 21;2(11):e0000747. doi: 10.1371/journal.pgph.0000747 (PMC10022348; doi:10.1371/journal.pgph.0000747)
Supplement: S4 Fig — (DOCX) [file pgph.0000747.s004.docx]

**
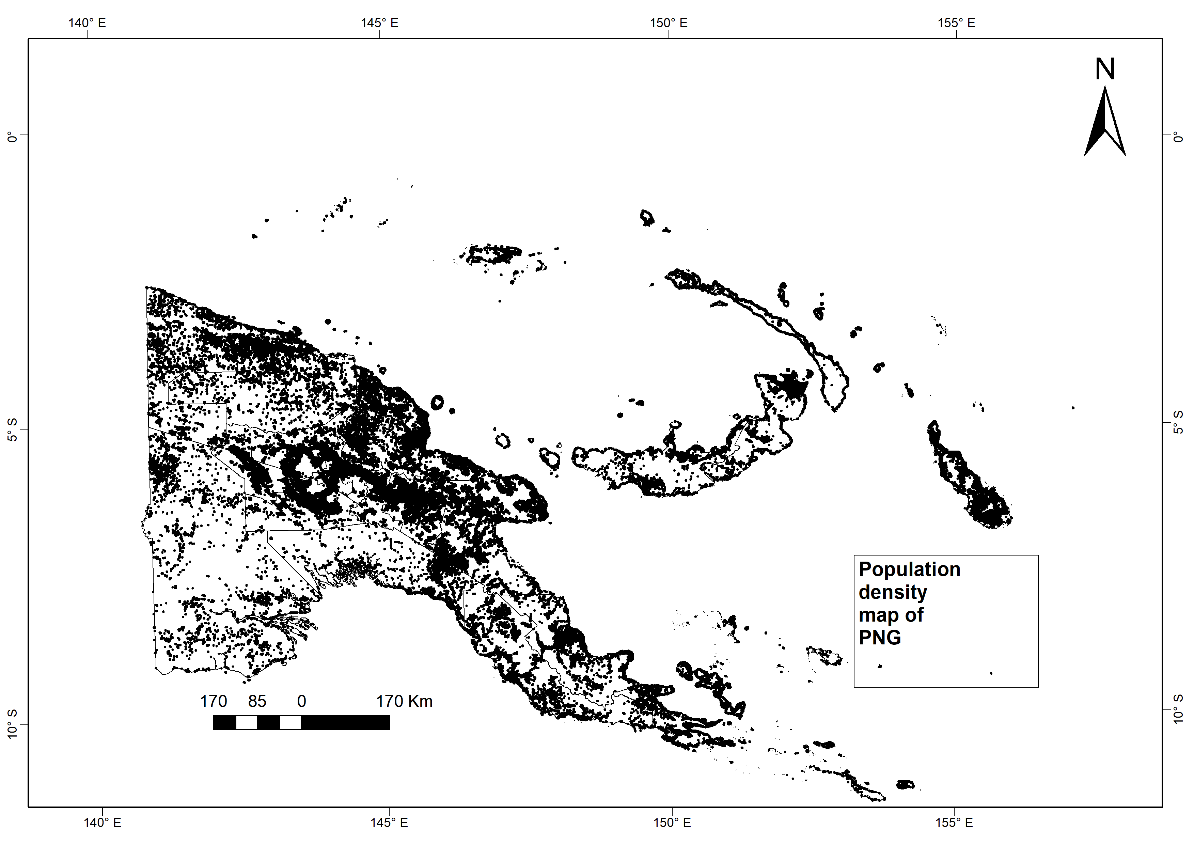
**

**S4 Fig. a) Population density map of PNG.** Data source: Facebook-Columbia University ([https://dataforgood.fb.com](https://dataforgood.fb.com)).


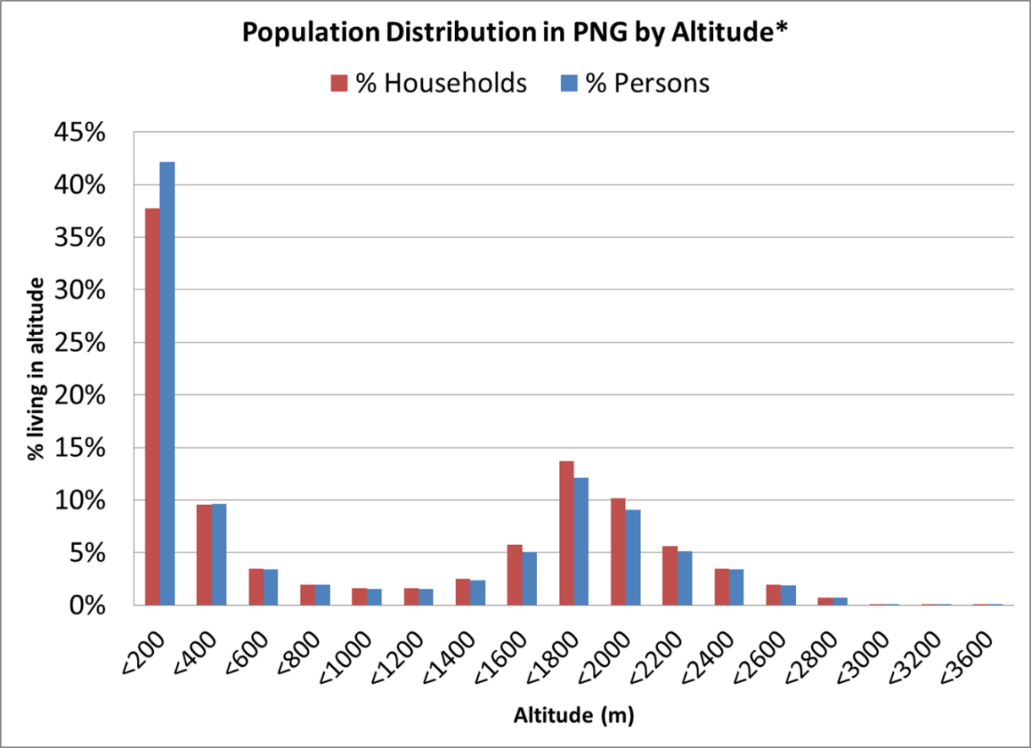


**S4 Fig. b)** Population distribution in PNG 2011 by altitude**.** Percentages of population and households in PNG by altitude. *Average altitude of census units in a radius of one km. Data source: 2011 National Population and Housing Census, NSO-PNG.


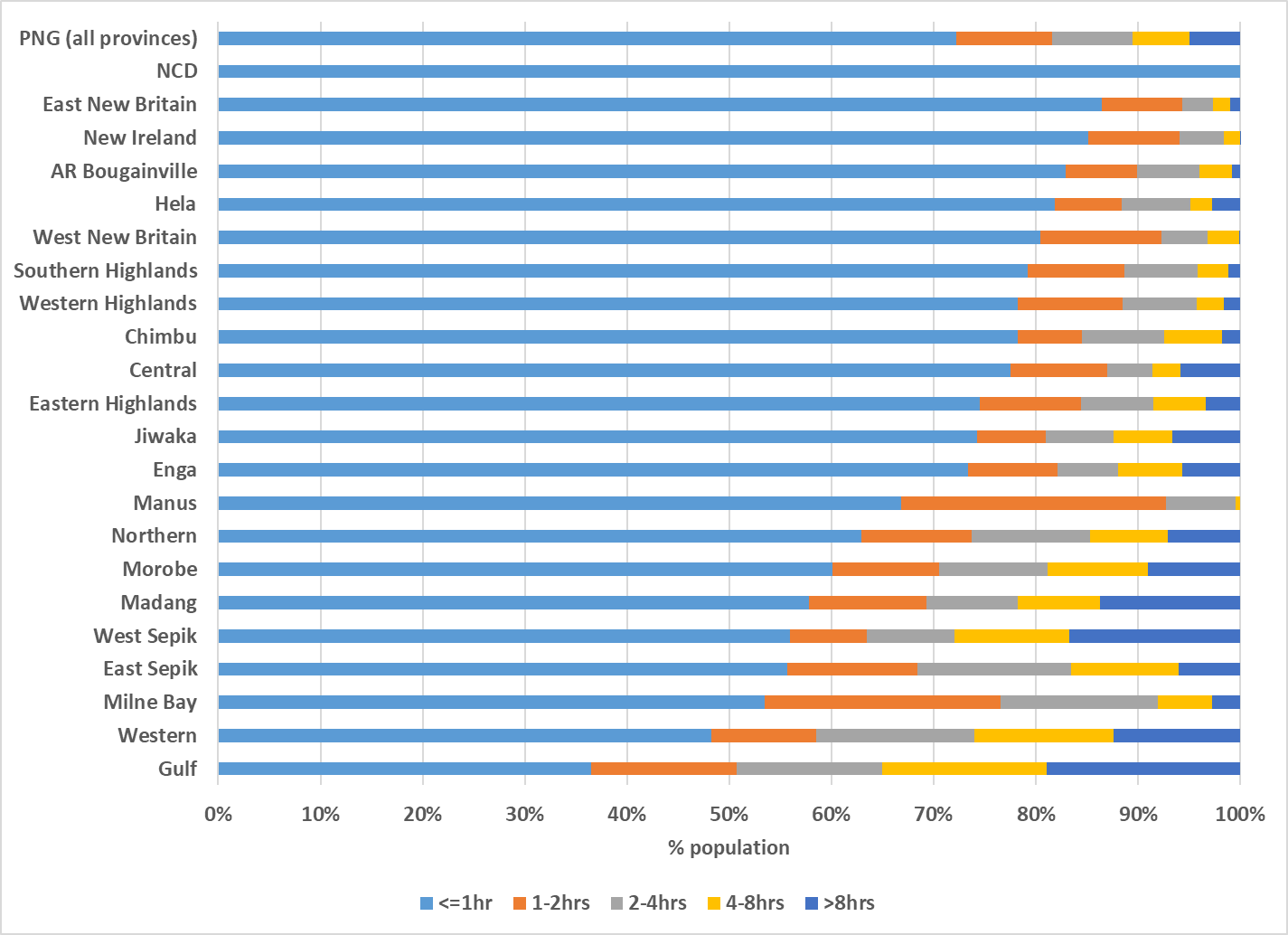


**S4 Fig. c)** Population distribution by travel time (hours) to the nearest health facility, by province.
