# Supplementary figures and images for: Stratification of malaria incidence in Papua New Guinea (2011–2019): Contribution towards a sub-national control policy
Source: PLOS Glob Public Health. 2022 Nov 21;2(11):e0000747. doi: 10.1371/journal.pgph.0000747 (PMC10022348; doi:10.1371/journal.pgph.0000747)

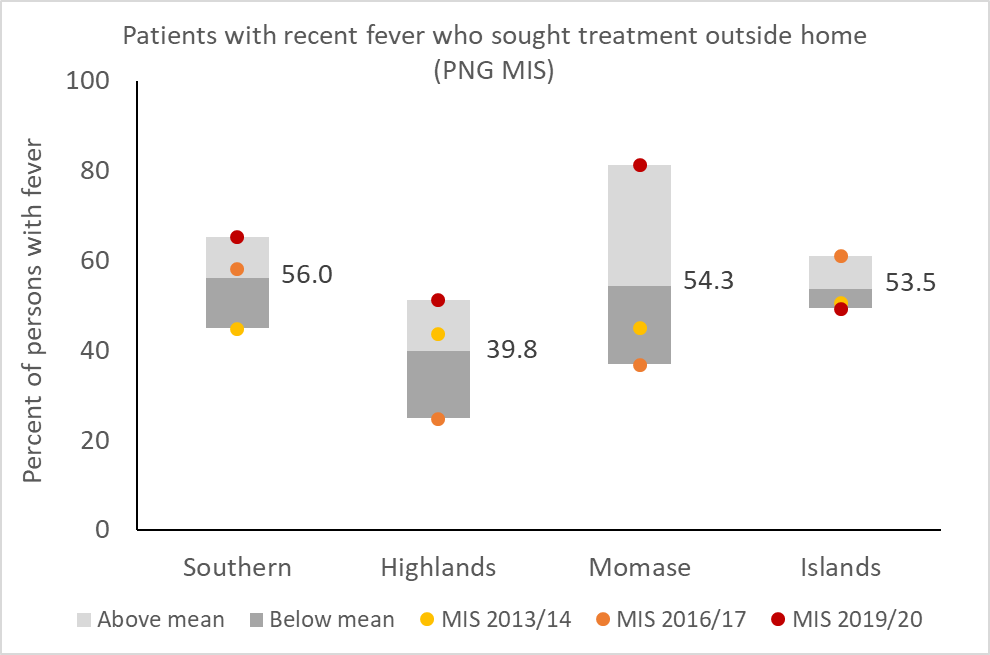

Supplement: S5 Fig — (TIF) [file pgph.0000747.s005.tif]

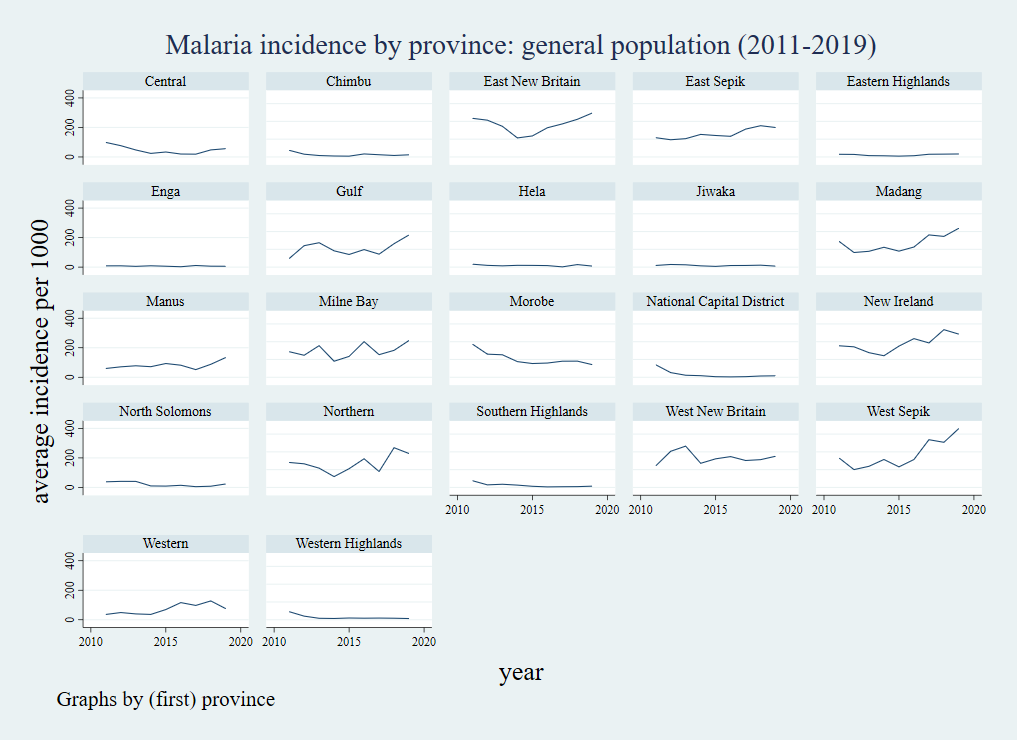

Supplement: S6 Fig — (TIF) [file pgph.0000747.s006.tif]

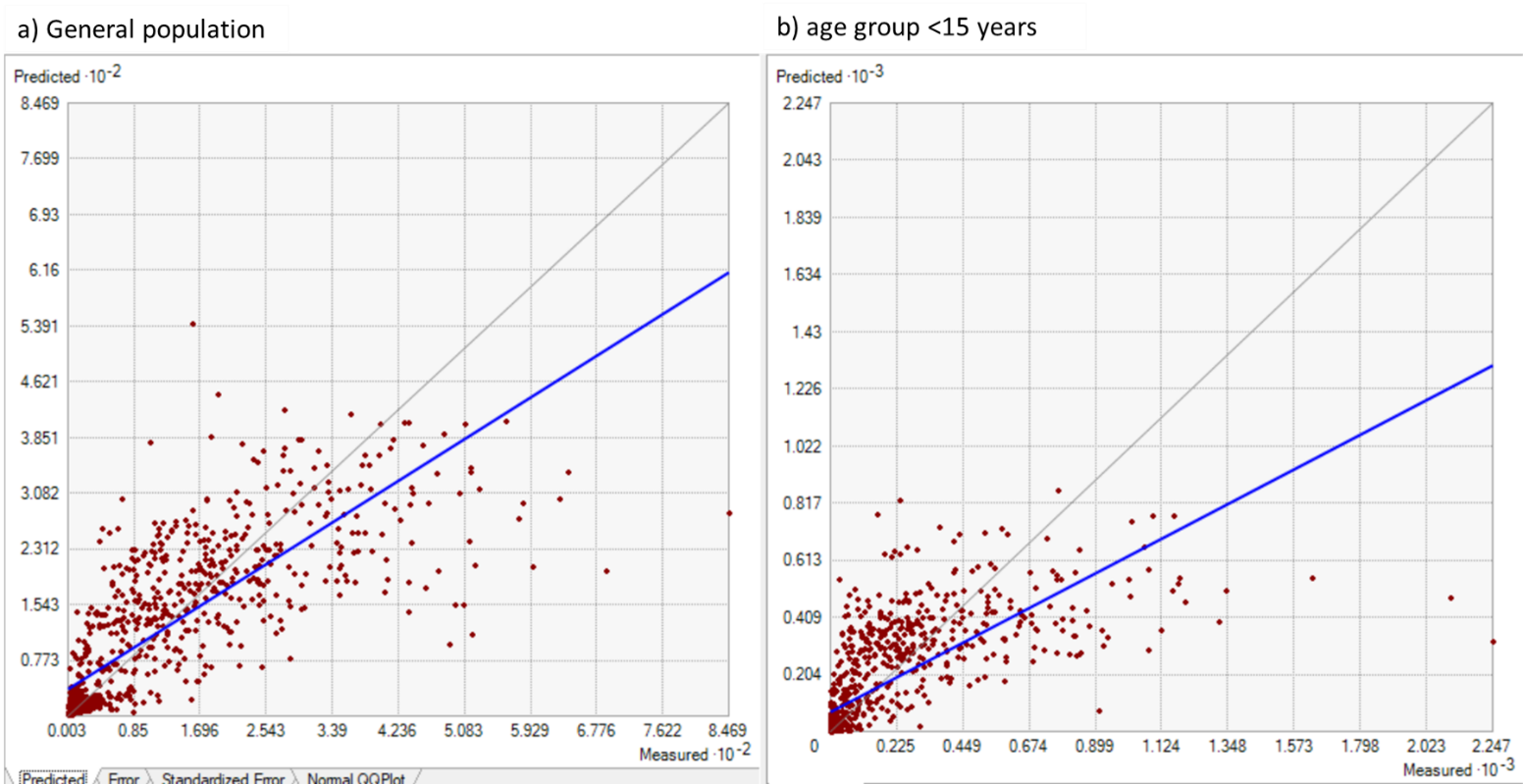

Supplement: S7 Fig — (TIF) [file pgph.0000747.s007.tif]
