# Supplementary material for: Stratification of malaria incidence in Papua New Guinea (2011–2019): Contribution towards a sub-national control policy
Source: PLOS Glob Public Health. 2022 Nov 21;2(11):e0000747. doi: 10.1371/journal.pgph.0000747 (PMC10022348; doi:10.1371/journal.pgph.0000747)
Supplement: S1 Text — (DOCX) [file pgph.0000747.s008.docx]

**S1 Text.** Statistics of cross-validation of EBK models

From documentation on Cross-validation of empirical Bayesian Kriging models, Geostatistical Analyst, ArcGIS 10.6 (accessed 10/27/2021)

- - Mean Error—the averaged difference between the measured and the predicted values.

- - Root Mean Square Error—indicates how closely your model predicts the measured values. The smaller this error, the better.

- - Average Standard Error—The average of the prediction standard errors.

- - Mean Standardised Error—The average of the standardised errors. This value should be close to 0.

- - Root Mean Square Standardized Error—This should be close to 1 if the prediction standard errors are valid. If the root-mean-squared standardised error is greater than 1, you are underestimating the variability in your predictions. If the root-mean-square-standardised error is less than 1, you are overestimating the variability in your predictions.
