# Supplementary material for: Stratification of malaria incidence in Papua New Guinea (2011–2019): Contribution towards a sub-national control policy
Source: PLOS Glob Public Health. 2022 Nov 21;2(11):e0000747. doi: 10.1371/journal.pgph.0000747 (PMC10022348; doi:10.1371/journal.pgph.0000747)
Supplement: S1 Table — Min-Max values are in parentheses. (DOCX) [file pgph.0000747.s009.docx]

**S1 Table.** Characteristics of health facilities (HFs): Averages of population, altitude, travel time and travel distance. Min-Max values are in parentheses

| **Province** | **No. HFs** | **Population** | **Altitude (m)** | **Travel time**  **(minutes)** | **Travel distance**  **(m)** |
| --- | --- | --- | --- | --- | --- |
| Bougainville | 38 | 7253  (1345, 19782) | 204  (10, 987) | 28  (18, 69) | 3520  (1206, 5192) |
| East New Britain | 32 | 10809  (1252, 30113) | 274  (14, 1078) | 31  (13, 68) | 3681  (2018, 5181) |
| Manus | 13 | 6048  (967, 24580) | 80  (18, 286) | 44  (12, 82) | 2915  (492, 5063) |
| New Ireland | 32 | 9186  (812, 45426) | 109  (12, 321) | 31  (17, 63) | 3341  (100, 4828) |
| West New Britain | 38 | 9015  (1291, 25029) | 128  (6, 339) | 36  (12, 79) | 3077  (889, 5043) |
| Central | 41 | 7789  (1118, 26411) | 497  (33, 2036) | 27  (12, 91) | 3063  (134, 5019) |
| Gulf | 21 | 7598  (1422, 22159) | 379  (16, 1658) | 44  (11, 94) | 3321  (1613, 5512) |
| Milne Bay | 44 | 7268  (1299, 20185) | 185  (20, 1378) | 46  (13, 80) | 3351  (100, 5683) |
| National Capital District | 27 | 18335  (3680, 100617) | 85  (7, 193) | 1  (10, 5) | 899  (100, 2945) |
| Northern | 20 | 9840  (1441, 27860) | 368  (54, 1273) | 41  (16, 74) | 3777  (2274, 5308) |
| Western | 42 | 5824  (846, 40697) | 213  (16, 1832) | 34  (12, 72) | 2533  (247, 4906) |
| Chimbu | 36 | 17337  (1038, 50373) | 1947  (894, 2810) | 31  (14, 117) | 3040  (1229, 5232) |
| Eastern Highlands | 37 | 19573  (1378, 43677) | 1830  (1371, 2334) | 35  (11, 86) | 3818  (761, 6086) |
| Enga | 42 | 15608  (1390, 68922) | 2363  (1420, 2845) | 36  (16, 77) | 3011  (1254, 4655) |
| Hela | 36 | 19302  (1166, 62816) | 1839  (704, 2793) | 20  (13, 58) | 3004  (845, 7312) |
| Jiwaka | 28 | 15026  (2074, 48221) | 1566  (102, 2290) | 29  (18, 54) | 2901  (1561, 4081) |
| Southern Highlands | 48 | 16220  (1595, 68162) | 1668 (572, 2376) | 28  (14, 80) | 3353  (65, 7608) |
| Western Highlands | 46 | 10690  (1295, 43506) | 1977  (721, 3032) | 30  (11, 88) | 2594  (798, 4369) |
| East Sepik | 49 | 11026  (1168, 25260) | 199 (4, 455) | 35  (11, 92) | 3368  (100, 5397) |
| Madang | 48 | 11533  (1144, 35698) | 398  (56, 2519) | 36  (11, 82) | 3713  (908, 7300) |
| Morobe | 53 | 16138  (1128, 149606) | 818  (80, 2046) | 36  (11, 77) | 3430  (845, 5010) |
| West Sepik | 37 | 9076  (1046, 61056) | 475  (100, 1896) | 36  (17, 93) | 3453  (459, 6416) |
