# Supplementary material for: Stratification of malaria incidence in Papua New Guinea (2011–2019): Contribution towards a sub-national control policy
Source: PLOS Glob Public Health. 2022 Nov 21;2(11):e0000747. doi: 10.1371/journal.pgph.0000747 (PMC10022348; doi:10.1371/journal.pgph.0000747)
Supplement: S2 Table — (DOCX) [file pgph.0000747.s010.docx]

**S2 Table.** Parameters used in EBK models for stratification of malaria incidence in general population and age group under 15 years.

| **Input dataset** | Incidence among the general population | Incidence among age group < 15 years |
| --- | --- | --- |
| **Subset Size** | 30 | 30 |
| **Overlap Factor** | 1.2 | 1.1 |
| **Number of Simulations** | 100 | 100 |
| **Transformation** | Empirical | Empirical |
| **Semivarigoram Type** | K-Bessel | Exponential |
| **Neighbourhood type** | Standard Circular | Standard Circular |
| **Maximum neighbours** | 7 | 9 |
| **Minimum neighbours** | 3 | 3 |
| **Sector type** | - | 4 Sectors with 45° offset |
| **Angle** | - | 0 |
| **Radius (ha)** | 5.98 | 9.80 |
